# Distinct neurodevelopmental and epileptic phenotypes associated with gain- and loss-of-function *GABRB2* variants

**DOI:** 10.1016/j.ebiom.2024.105236

**Published:** 2024-07-11

**Authors:** Nazanin Azarinejad Mohammadi, Philip Kiær Ahring, Vivian Wan Yu Liao, Han Chow Chua, Sebastián Ortiz de la Rosa, Katrine Marie Johannesen, Yael Michaeli-Yossef, Aline Vincent-Devulder, Catherine Meridda, Ange-Line Bruel, Alessandra Rossi, Chirag Patel, Joerg Klepper, Paolo Bonanni, Sara Minghetti, Marina Trivisano, Nicola Specchio, David Amor, Stéphane Auvin, Sarah Baer, Pierre Meyer, Mathieu Milh, Vincenzo Salpietro, Reza Maroofian, Johannes R. Lemke, Sarah Weckhuysen, Palle Christophersen, Guido Rubboli, Mary Chebib, Anders A. Jensen, Nathan L. Absalom, Rikke Steensbjerre Møller

**Affiliations:** a Department of Epilepsy Genetics and Personalized Treatment, Danish Epilepsy Centre, Filadelfia (Member of the ERN EpiCARE), Dianalund, Denmark; b Department of Regional Health Research, University of Southern Denmark, Odense, Denmark; c School of Medical Sciences, Faculty of Medicine and Health, Brain and Mind Centre, The University of Sydney, Sydney, New South Wales 2006, Australia; d Sydney Pharmacy School, Faculty of Medicine and Health, Charles Perkins Centre, The University of Sydney, Sydney, New South Wales 2006, Australia; e Department of Genetics, University Hospital of Copenhagen, Rigshospitalet, Copenhagen, Denmark; f Pediatric Neurology Unit and Metabolic Neurogenetic Clinic, Wolfson Medical Center, Holon, Israel; g Genetic Department, CHU Côte de Nacre, Caen, France; h Pediatric Clinic, IRCCS San Matteo Hospital Foundation, University of Pavia, Pavia, Italy; i Genetic Health Queensland, Royal Brisbane & Women’s Hospital, Brisbane, QLD 4029, Australia; j Children's Hospital Aschaffenburg-Alzenau, Aschaffenburg, Germany; k IRCCS E. Medea Scientific Institute, Epilepsy Unit, Conegliano, Treviso, Italy; l IRCCS E. Medea Scientific Institute, Clinical Neurophysiology Unit, Bosisio Parini, LC, Italy; m Neurology, Epilepsy and Movement Disorders, Bambino Gesù Children’s Hospital, IRCCS, Full Member of European Reference Network EpiCARE, Rome, Italy; n Murdoch Children’s Research Institute, Melbourne, Australia; o Université de Paris, Child Neurology & Epilepsy, Paris, France; p Robert-Debré Hospital, Center for Rare Epilepsies - Pediatric Neurology, Paris, France; q Department of Paediatric Neurology, French Reference Center of Rare Epilepsies CREER, Hôpitaux Universitaires de Strasbourg, Strasbourg, France; r Paediatric Neurology Department, Phymedexp, Montpellier University, Inserm, CNRS, University Hospital Montpellier, Montpellier, France; s Department of Pediatric Neurology, AP-HM, La Timone Children's Hospital, Marseille, France; t Faculté de Médecine Timone, Aix Marseille Univ, INSERM, MMG, U1251, ERN EpiCARE, Marseille, France; u Department of Neurosciences Rehabilitation, Ophthalmology, Genetics, Maternal and Child Health (DiNOGMI), University of Genoa, Genoa, Italy; v Pediatric Neurology and Muscular Diseases Unit, IRCCS Giannina Gaslini Institute, Genoa, Italy; w Department of Neuromuscular Disorders, UCL Queen Square Institute of Neurology, London, UK; x Institute of Human Genetics, University of Leipzig Medical Center, Leipzig, Germany; y Center for Rare Diseases, University of Leipzig Medical Center, Leipzig, Germany; z Applied & Translational Neurogenomics Group, VIB Center for Molecular Neurology, VIB, Antwerp, Belgium; aa Department of Neurology, Antwerp University Hospital, Antwerp, Belgium; ab Translational Neurosciences, Faculty of Medicine and Health Science, University of Antwerp, Antwerp, Belgium; ac Saniona A/S, Ballerup, Denmark; ad Department of Clinical Medicine, Faculty of Health and Medical Sciences, University of Copenhagen, Copenhagen, Denmark; ae Department of Drug Design and Pharmacology, Faculty of Health and Medical Sciences, University of Copenhagen, Copenhagen, Denmark; af School of Science, Western Sydney University, Sydney, Australia

**Keywords:** GABA_A_ receptors, Gain-of-function, Epilepsy, Seizures, Dystonia, Movement disorders

## Abstract

**Background:**

Variants in *GABRB*2, encoding the β2 subunit of the γ-aminobutyric acid type A (GABA_A_) receptor, can result in a diverse range of conditions, ranging from febrile seizures to severe developmental and epileptic encephalopathies. However, the mechanisms underlying the risk of developing milder vs more severe forms of disorder remain unclear. In this study, we conducted a comprehensive genotype–phenotype correlation analysis in a cohort of individuals with *GABRB*2 variants.

**Methods:**

Genetic and electroclinical data of 42 individuals harbouring 26 different *GABRB*2 variants were collected and accompanied by electrophysiological analysis of the effects of the variants on receptor function.

**Findings:**

Electrophysiological assessments of α1β2γ2 receptors revealed that 25/26 variants caused dysfunction to core receptor properties such as GABA sensitivity. Of these, 17 resulted in gain-of-function (GOF) while eight yielded loss-of-function traits (LOF). Genotype-phenotype correlation analysis revealed that individuals harbouring GOF variants suffered from severe developmental delay/intellectual disability (DD/ID, 74%), movement disorders such as dystonia or dyskinesia (59%), microcephaly (50%) and high risk of early mortality (26%). Conversely, LOF variants were associated with milder disease manifestations. Individuals with these variants typically exhibited fever-triggered seizures (92%), milder degrees of DD/ID (85%), and maintained ambulatory function (85%). Notably, severe movement disorders or microcephaly were not reported in individuals with loss-of-function variants.

**Interpretation:**

The data reveals that genetic variants in *GABRB*2 can lead to both gain and loss-of-function, and this divergence is correlated with distinct disease manifestations. Utilising this information, we constructed a diagnostic flowchart that aids in predicting the pathogenicity of recently identified variants by considering clinical phenotypes.

**Funding:**

This work was funded by the Australian National Health & Medical Research Council, the 10.13039/501100009708Novo Nordisk Foundation and The 10.13039/501100003554Lundbeck Foundation.

**Research in context**

**Evidence before this study**

GABA_A_ receptors, which serve as the primary inhibitory ligand-gated ion channels in the mammalian brain, play a crucial role in regulating essential neurophysiological functions such as movement, learning, and memory processes. Variants in the *GABRB*2 gene, which encodes the GABA_A_ receptor β2 subunit, have been implicated in a broad range of neurodevelopmental disorders, epilepsies, and movement disorders. Despite their significance, the underlying pathophysiology remains poorly understood. Traditionally, it was believed that *GABRB*2 variants primarily led to loss of receptor function, resulting in hyperexcitation in neuronal networks and subsequent epilepsy. However, recent research has revealed that not only loss-of-function but also gain-of-function variants in other GABA_A_ receptor subunits can contribute to severe epilepsy. Therefore, we hypothesised that the lack of understanding regarding *GABRB*2 variant pathophysiology may stem from divergent functional consequences.

**Added value of this study**

We performed molecular and clinical analyses on 26 missense variants in the *GABRB*2 gene, identified from 42 individuals with neurodevelopmental disorders. Our findings shed light on the impact of gain-of-function *GABRB*2 variants, which can lead to catastrophic early onset epilepsies, severe intellectual disability, movement disorders and high risk of early death. Interestingly, we observed that the severity of clinical outcomes correlates with the degree of functional changes induced by these gain-of-function variants. By contrast, milder forms of neurodevelopmental disorders and epilepsies, particularly those with fever sensitivity, were associated with loss-of-function variants. These findings highlight the importance of considering both loss- and gain-of-function *GABRB*2 variants in the context of neurodevelopmental disorders and epilepsy.

**Implications of all the available evidence**

Understanding the functional consequences of genetic variants is essential for improving clinical outcomes, including accurate diagnosis, effective counselling, and ideally targeted treatment. While efficient therapies should alleviate symptoms and potentially reverse specific variant-induced functional changes, it is equally crucial to avoid treatments that might exacerbate a patient’s condition by worsening the underlying molecular defect. Based on our extensive molecular and clinical data, we have developed a diagnostic flowchart that utilises clear clinical biomarkers to predict the pathogenicity of newly identified *GABRB*2 variants. This tool will be useful in improving diagnosis and achieving precision medicine for future patients with *GABRB*2 variants.

## Introduction

γ-Aminobutyric acid type A receptor (GABA_A_ receptor)-associated neurodevelopmental disorders are clinically challenging to diagnose/treat due to the wide spectrum of encephalopathies and epilepsies that differ in seizure types and severity of disease progression.^1^, ^2^, ^3^, ^4^, ^5^, ^6^, ^7^, ^8^, ^9^, ^10^, ^11^, ^12^, ^13^ The associated syndromes can range from simple febrile seizures, genetic epilepsy with febrile seizures plus (GEFS+) or genetic generalised epilepsies (GGE) to severe developmental and epileptic encephalopathies (DEEs) such as epilepsy of infancy with migrating focal seizures (EIMFS), infantile epileptic spasms syndrome (IESS), Dravet syndrome and Lennox-Gastaut syndrome (LGS).^14^^,^^15^ Importantly, the mechanisms underlying this phenotypic diversity and risks of developing severe co-morbidities such as the prominent movement disorders associated with *GABRB*2, are unclear.^1^

GABA_A_ receptors are ligand-gated ion channels that mediate neuronal inhibition by allowing chloride influx in response to GABA activation. Structurally, GABA_A_ receptors are pentameric assemblies with large subtype heterogeneity driven by 19 different subunit genes. However, the majority of GABA_A_ receptors in mammalian brain contain two α subunits, two β subunits, and a γ or δ subunit.^16^, ^17^, ^18^ Of the three β subunits, the β2 and β3 subunits encoded by *GABRB*2 and *GABRB3* genes constitute the bulk of total β subunit protein levels,^19^ and are both expressed in early development with a largely overlapping spatial distribution pattern in the adult brain.^20^ Notably, clinical phenotypes associated with *GABRB2* and *GABRB3* variants have a spectrum of largely similar features.^1^^,^^11^

In recent studies, the phenotypic spectrum described for individuals with variants in the *GABRB2* gene included neurodevelopmental and epileptic phenotypes from milder forms of within the GEFS+ spectrum to severe forms of DEE.^1^^,^^8^ Notably, nearly half of one cohort exhibited comorbid severe movement disorders, including dystonia, dyskinesia, hyperkinesia, and chorea.^1^ While functional evaluation of a limited number of variants implicated loss-of-function (LOF) traits as the underlying pathomechanism, this analysis fell short of explaining the heterogeneity of the syndromes or associated comorbidities.^1^ Moreover, recent research has challenged the prevailing notion that LOF variants solely account for the entire clinical spectrum. These studies have revealed that both gain-of-function (GOF) and LOF variants in not only the *GABRB3* gene but also the *GABRA1*, *GABRA4,* and *GABRD* genes are associated with distinct clinical phenotypes in individuals with DEE.^9^^,^^11^^,^^13^^,^^21^, ^22^, ^23^ Considering the overlapping distribution of β2 and β3 subunits and the phenotypic similarity of individuals with *GABRB2* and *GABRB3* variants, we hypothesised that individuals with GOF and LOF variants in the *GABRB2* gene may similarly segregate into distinct clinical sub-cohorts.

In this study, we assembled a cohort of 42 individuals harbouring 26 presumed pathogenic *GABRB2* variants, which included both unpublished and previously reported individuals. Comprehensive functional analysis was performed for all 26 variants using electrophysiological recordings from α1β2γ2 receptors. These analyses revealed distinct functional receptor categories, including both GOF and LOF variants. Importantly, we identified clear distinctions in clinical manifestations between individuals with GOF and LOF variants. These findings facilitated the development of a diagnostic flowchart, which can be used to predict the variant type for *GABRB2*-associated epilepsies and related diseases.

## Methods

### Clinical ascertainment

Individuals with presumed pathogenic variants in *GABRB2* were included for clinical and functional characterisation. Our cohort included a total of 42 individuals; 13 unreported, 8 previously published for whom additional clinical information was available and 21 from the literature.^1^^,^^8^^,^^24^, ^25^, ^26^, ^27^ The previously uncharacterised individuals were recruited through an international network of epilepsy and genetic centres in Europe as well as via the European Reference Network (ERN) ERN-EpiCare Genetic Platform (https://epi-care.eu/collaborative-genetic-research/). Demographic, genetic and clinical information on early developmental milestones, cognition, age at seizure onset, seizure types, epilepsy syndrome, electroencephalogram (EEG) and Magnetic Resonance Imaging (MRI) findings, current treatment, movement disorders, neuro-psychiatric/behavioural features and information on early mortality (before 18 years of age) was collected by face-to-face interviews with individuals and their families or from detailed review of medical records. Inclusion criteria for previously published individuals included availability of detailed clinical information and an emphasis on variants located in the transmembrane domain of the β2 subunit. It was previously shown that variants in the transmembrane domain of the β3 subunit cause a more severe phenotype compared to those in the extracellular domain^28^ and have a high likelihood of causing GOF.^11^ Hence such variants were prioritised for *GABRB2* to increase the probability of identifying sufficient GOF variants to ensure a detailed description of the clinical phenotype. All data were collected in a structured phenotype table hosted at the Danish Epilepsy Centre. The epilepsy syndromes were classified according to the most recent ILAE classification.^29^^,^^30^ Data are reported in line with the Strengthening Reporting of Observational Studies in Epidemiology (STROBE) statement.

### Classification and structural mapping of *GABRB2* variants

The genetic findings in the unpublished cohort were obtained through routine diagnostic testing with either a targeted gene panel or whole exome sequencing. The *GABRB2* variants collected from the literature were found by targeted next generation sequencing epilepsy panels or whole exome sequencing performed either in routine diagnostic or research settings.^1^^,^^8^^,^^24^, ^25^, ^26^, ^27^ All 26 *GABRB2* variants were annotated using transcript NM_001371727 and assessed using SIFT (sorting intolerant from tolerant), PolyPhen-2 (polymorphism phenotyping-v2) and CADD v1.6 (combined annotation dependent depletion). Variants were classified according to the American College of Medical Genetics and Genomics guidelines.^31^ With the exception of R293W, which had a single entry, all variants were absent from the control database gnomAD v4.0.0 (genome aggregation database). The gnomAD database consist of exome and genome sequences from individuals without paediatric disease and serve as a very useful reference sets of allele frequencies for severe paediatric disease studies.

**Fig. 1:**
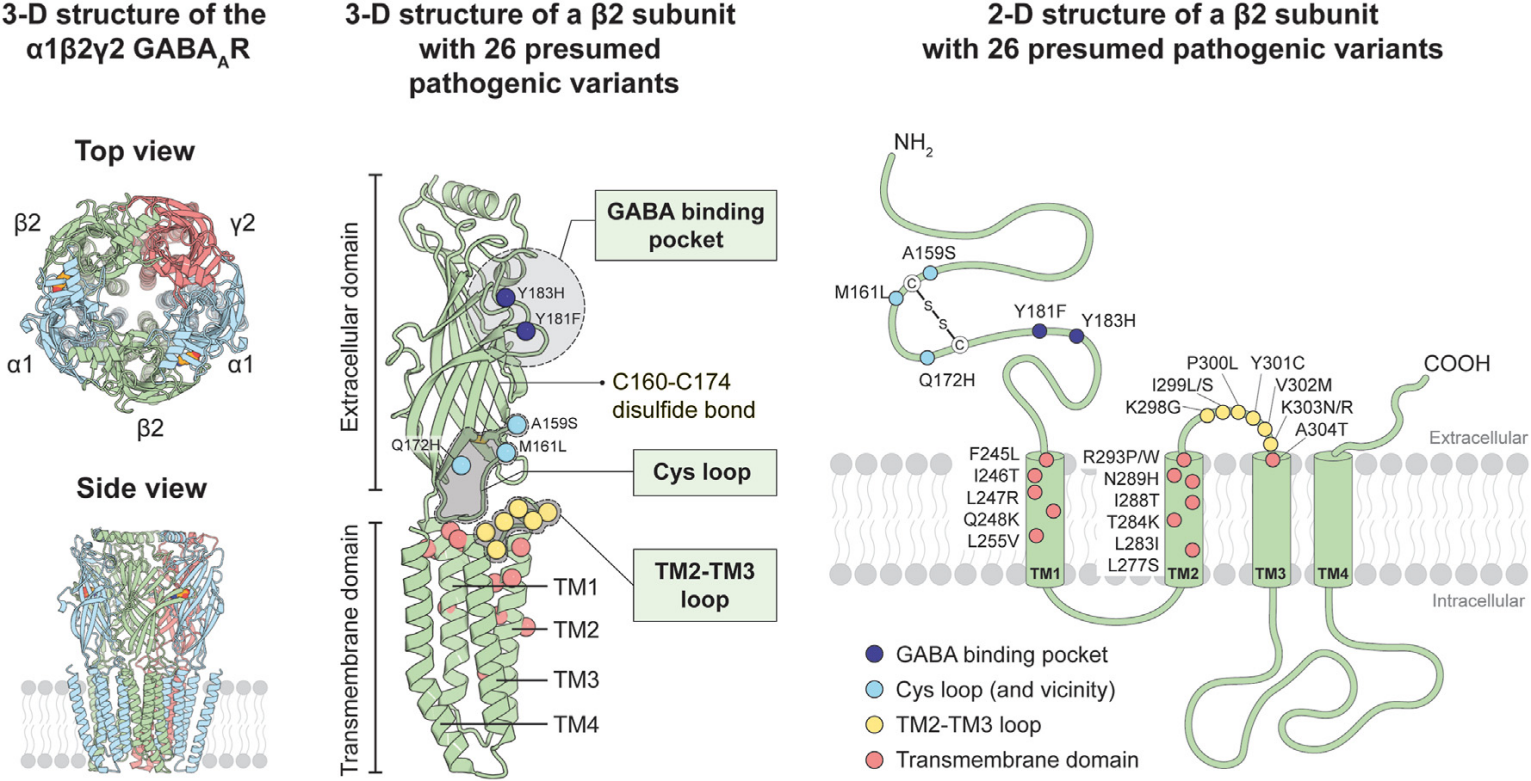
Missense *GABRB2* variants associated with epilepsy or neurodevelopmental disorders. (**Left**) 3-D structure of a GABA_A_ receptor is adapted from the cryo-electron microscopy (Cryo-EM) structure of the pentameric α1β2γ2 GABA_A_ receptor (6x3z.pdb). (**Middle**) 3-D structure of β2 subunit illustrating the location of 26 presumed pathogenic variants as spheres. Variants are enriched in three important functional regions: GABA binding pocket, coupling region (Cys-loop and TM2-TM3 loop) and transmembrane helices. (**Right**) Membrane topology of the GABA_A_ receptor β2 subunit with spheres representing the relative location of 26 individual missense *GABRB2* variants. Variants are colour-coded according to the functional region within the subunit: GABA binding pocket (dark blue), Cys-loop and residues in proximity (light blue), TM2-TM3 loop (yellow), and transmembrane helices TM1-TM3 (pink). Each variant is indicated by line pointing its position and amino acid substitution.

All variants were found in regions characterised by a high degree of conservation across subunits of the GABA_A_ receptor family (Fig. 1). Nine of the amino acid residues affected are fully conserved across the α1-6, β1-3 and γ1-3 subunits (Y181, Y183, F245, Q248, L283, T284, R293, Y301 and A304), while the remaining 14 residues are conserved within the three β subunits. Five out of the 26 variants are located in the extracellular domain of β2: Y181F and Y183H are in the GABA binding pocket; A159S and M161L flank C160, one of the two critical cysteine residues forming the signature Cys-loop via a disulfide bond; and Q172H is within the Cys-loop itself. Twenty-one variants are located in the transmembrane domain of β2 (Fig. 1). Eight of these are in the linker between M2 and M3, a region known to interact with a several extracellular regions, including the Cys-loop in the coupling region translating the GABA binding event into channel gating. The remaining 13 variants are located in the transmembrane helices M1-M3 that contribute to forming the ion channel pore (Fig. 1). Hence, all 26 variants in this study reside in regions known to be essential for receptor function.^17^

### Molecular biology

The design of concatenated pentameric receptor constructs using human GABA_A_ receptor subunits has previously been described.^32^, ^33^, ^34^ For this study, a tetrameric γ2-X-α1-β2-α1 construct in which X represents a “missing” β2-subunit position was applied to allow for systematic introduction of a point-mutated β2-subunit in only one of the two β2-subunit positions in the α1β2γ2 (γ2-β2-α1-β2-α1) pentamer. The 26 β2-subunit mutations were made and verified by sequencing followed by sub-cloning into the concatenated construct using standard restriction digestion and ligation. Linearised cDNA was generated and cRNA for each concatenated receptor construct was produced using the mMessage mMachine T7 Transcription Kit (Thermo Fisher).

### *Xenopus laevis* oocytes

Oocytes were purchased from Oocyte Bioscience. The cRNAs of wildtype and the 26 mutant a concatenated α1β2γ2 receptors were injected into oocytes at ∼25 ng cRNA per oocyte. Then oocytes were incubated for 2 days at 18 °C in modified Barth’s solution (96 mM NaCl, 2 mM KCl, 1 mM MgCl_2_, 1.8 mM CaCl_2_, 5 mM 4-(2-hydroxyethyl)-1-piperazineethanesulfonic acid (HEPES), 2.5 mM sodium pyruvate, 0.5 mM theophylline, and 100 mg/L gentamicin; pH 7.4).

### Electrophysiology

Electrophysiological recordings of GABA concentration-response relationships and maximal GABA-evoked current amplitudes for wildtype and mutant α1β2γ2 receptors were performed using a custom made two-electrode voltage clamp apparatus described previously.^11^^,^^33^^,^^35^ All recordings were performed at room temperature. Briefly, oocytes were placed in a recording chamber, and a saline solution termed OR2 (90 mM NaCl, 2.5 mM KCl, 2.5 mM CaCl_2_, 1 mM MgCl_2_ and 5 mM HEPES; adjusted to pH 7.4 with HCl) was continuously perfused. The pipettes were backfilled with 3 M KCl and had open pipette resistances from 0.4 to 2 MΩ when submerged in OR2 solution. Oocytes were voltage clamped using an Axon GeneClamp 500 B amplifier (Molecular Devices) at a holding potential of −60 mV. Amplified currents were low-pass filtered at 20 Hz using a four-pole Bessel filter (Axon GeneClamp 500 B), digitised using a Digidata 1322 B (Molecular Devices) and sampled at 200 Hz on a personal computer using the pClamp 10.2 suite (Molecular Devices). Episodic traces following triggering events representing responses to individual applications were collected.

For desensitisation experiments, another setup with ultra-low bath volumes was used to ensure rapid liquid exchange.^36^ 3 M KCl-filled borosilicate glass microelectrodes with resistance of 0.2–1.6 MΩ were inserted into cells then clamped at −60 mV with constant perfusion of ND96 solution (96 mM NaCl, 2 mM KCl, 1 mM MgCl_2_, 5 mM HEPES, 1.8 mM CaCl_2_, pH 7.4) through a gravity-driven semi-automatic system at 1 mL/min. A Warner OC-725C amplifier (Warner Instruments) was used for amplifying GABA-evoked currents that were then filtered and digitised at 10 Hz using the Powerlab 8/35 with LabChart reader version 8.1 (AD Instruments).

### Experimental protocols

On each experimental day, the functional properties of wildtype α1β2γ2 receptors were assessed along with the mutant receptors to eliminate the impact of inter-day variation and variation between batches of oocytes. To assess maximum current amplitudes, 10 mM GABA was applied, and final datasets for this parameter consisted of at least 22 independent experiments performed on at least two different batches of oocytes. A series of control applications were performed prior to the GABA concentration-response experiments to ensure reproducibility of evoked amplitudes. The control applications were: three GABA_control_ (2–100 μM; approximately EC_5-30_) applications, one GABA_max_ (316–10,000 μM; approximately EC_100_) application followed by another three GABA_control_ applications. The GABA concentration-response relationship was then determined by applications of increasing concentrations of GABA to the oocyte. Final datasets for GABA concentration-response were collected from at least 10 independent experiments performed on at least two different batches of oocytes.

Raw traces were analysed using pClamp 10.2 or LabChart reader version 8.1. To determine the EC_50_ values of GABA concentration-response relationships, the Hill equation was fitted to peak GABA-evoked current amplitudes for individual oocytes using least-squares estimation of nonlinear parameters^37^ in GraphPad Prism 8:

$$I={\mathrm{Abs}.I}_{\max}\left({\left[A\right]}^{nH}/\left({\left[A\right]}^{nH}\;+\;{\left[{\mathrm{EC}}_{50}\right]}^{nH}\right)\right)$$

Where $\text{Abs}.I_{\max}$ is the absolute maximum current, ${\text{EC}}_{50}$ is the concentration that evoke half-maximum response, [A] is the ligand (GABA) concentration and nH is the Hill slope. For each individual oocyte, a complete concentration-response curve was recorded as a single determination (n). From the ${\text{EC}}_{50}$ value the corresponding $\log\;{\text{EC}}_{50}$ value was calculated. By fitting the Hill equation to all data for each construct, final ${\text{EC}}_{50}$ values were calculated. For each experimental day the mean $\log\;{\text{EC}}_{50}$ for wildtype construct ($\log\;{\text{EC}}_{50,\text{wt}}$) was calculated. In addition, the $\Delta \log\;{\text{EC}}_{50}$ value for each oocyte containing a mutant construct tested on the same day was calculated using the following equation:

$$\Delta \log{\mathrm{EC}}_{50}=\log{\mathrm{EC}}_{50,wt}\;-\;\log{\mathrm{EC}}_{50}$$

The normalised maximum GABA-evoked current amplitude ($I_{\max}$) was calculated using the peak current evoked by 10 mM GABA at wildtype controls (${\text{Abs}.I}_{\max,\text{wt}}$) and mutants $\left({\text{Abs}.I}_{\max}\right)$ for parallel experiments performed on the same experimental day. To determine the $(I_{\max}$) for each individual experiment on a variant following equation was used:

$$I_{\max}=\frac{\mathrm{Abs}.I_{\max}}{{\mathrm{Abs}.I}_{\max,\mathrm{wt}}}$$

For desensitisation experiments, the recording protocol consisted of: a 2-min wash period with ND96 buffer, a 150-s application of 3 mM GABA, a 5-min wash period, another 150-s application of 3 mM GABA, an 8-min wash period, and finally a 120-s co-application of 10 mM GABA and 10 μM etomidate. The peak current amplitude induced by the second 3 mM GABA application was normalised to that induced by 10 mM GABA and 10 μM etomidate to calculate the maximum GABA-evoked receptor open probability (Est P_O(max)_).^36^ For desensitisation experiments, non-linear regression was performed with GraphPad Prism 8. The following equation was used to fit traces to one-phase exponential decay:

$$Y=\left(Y_{0}\;-\;\mathrm{Plateau}\right)\;\cdot \;e^{-kx}\;+\;\mathrm{Plateau}$$

Y represents current amplitude and x the time. The Plateau/asymptote of each fitted trace is the steady-state ($I_{\text{ss}}$) and k (s^−1^) represents the rate constant of current decay. To estimate the maximum steady-state open probability (${\text{Est}.\;P}_{O\left(\text{ss},\max\right)}$) the $I_{\text{ss}}$ was normalised to the ${\text{Est}.\;P}_{O\left(\max\right)}$.

### Statistics

For statistical comparison of GABA sensitivity measurements, the mean $\Delta \log{\text{EC}}_{50}$ for all mutants were calculated and presented as mean ± S.D. To prevent false positives of small but significant changes in GABA sensitivity from oversampling, a minimum threshold change was set at ±0.2 meaning that variants would need to give larger differences to be considered as significant. The value of ±0.2 corresponds to the standard deviation of the wildtype ΔlogEC_50_ value rounded to one decimal point. Statistical analysis was performed using One-way ANOVA with Dunnett’s corrected post hoc test with a *P* < 0.0001 threshold, and normality tests were performed to ensure that the logEC_50_ values conformed to a normal distribution.

For maximal current amplitude measurements, data are presented as median with interquartile ranges (IQR). Statistical comparison was made using a mean rank Mann–Whitney U test with a *P* < 0.0001 threshold to compare values for wildtype with mutant receptors for an equal number of experiments performed on the same experimental days. It is, however, difficult to gauge how a current loss observed in a heterologous expression system reflects changes in neurons, where compensatory mechanisms may alleviate many types of issues.^12^ Therefore, besides a statistical threshold of *P* < 0.0001, a minimum threshold change was defined at 0.5 (i.e., an I_max_ of 50% of the I_max_ of the wildtype receptor) to ensure that only mutants with substantial detriments to their functional expression level were assigned a LOF designation.

To compare the desensitisation properties exhibited by different constructs, One-way Analysis of Variance (ANOVA) (Kruskal Wallis rank sum test) followed by a Dunn’s post-hoc test was used to determine significance. The data for the mutants were compared to each other and to wildtype receptor data recorded on the same day. A minimum of two batches of oocytes were used to carry out the experiments for each construct and data are presented as mean ± S.D.

For the clinical data, the age of seizure onset was compared with a mean-rank Mann–Whitney U test and the variance was compared with an F-test, while the Mantel–Cox log-rank test was performed to account for individuals with no seizures. Mortality was compared with a Mantel–Cox log-rank test. For qualitative clinical outcomes including presence of severe intellectual disability, movement disorders (limited to including dystonia, dyskinesia, hyperkinesia and/or chorea), microcephaly, seizure freedom, hypotonia and fever-triggering seizures, the odds ratio was compared with Fisher’s Exact test as cell counts for some indications were likely to be zero. The Baptista-Pike Method was used to calculate 95% confidence intervals (CI) for Odds Ratio Estimates. All tests were performed with the software Graphpad Prism 9.0.

Survival and incidence times were censored at the last follow-up age. Where individuals were too young for a specific indication (e.g., movement disorder) or were not assessed for a specific indication, they were censored. This is indicated in the tables as “UK (unknown)” or “NR (not relevant)”.

### Ethics

The study was conducted according to the ethical principles for medical research outlined in the Declaration of Helsinki. The study was approved by the local ethics committee in the Zealand region of Denmark (number SJ-91), and by the Institutional Review Board at the Danish Epilepsy Centre, Filadelfia (EMN-2024-01998). Written or oral informed consent for participation was provided by parents or legal guardians, and the appropriate institutional forms have been archived.

### Role of funders

The funders had no role in study design, data collection, data analysis, interpretation or writing of the report.

## Results

### Genetic landscape

We collected a cohort of 42 individuals (18 females and 24 males) with neurodevelopmental disorders and epilepsy attributed to variants in the *GABRB2* gene (Supplementary Table S1). The 42 individuals harboured 26 variants that were heterozygous missense and occurred either *de novo* or segregated with the disease in one family (R293W). While 13 individuals are newly identified, 29 have previously been published, and we provide additional information for 8 of them. Two individuals were mosaic for their variants, L247R (30%) and I288T (degree unknown), and another variant, A304T, occurred presumably *de novo* in two paternal half-brothers, indicating that the father is mosaic for the variant. The father was diagnosed with epilepsy at 5 years of age, but no further information was available.

The 26 missense *GABRB2* variants selected caused alterations in 23 amino acid positions with two different variants observed at three residue positions (R293 P/W, I299 L/S, K303 N/R) (Fig. 1). Recurrent variants were seen for Y181F, I246T, L277S, R293P, Y301C, V302M, K303N, K303R and A304T. All variants were absent from the general population (gnomAD). With the exception of A159S, all were predicted to be damaging by PolyPhen-2 and/or SIFT and had a CADD score between 22.9 and 32, which suggest a high likelihood of deleteriousness (Supplementary Table S1). A159S had a CADD score of 23.6 but was predicted to be tolerated by both PolyPhen-2 and SIFT.

### Functional analysis of *GABRB2* variants

**Fig. 2:**
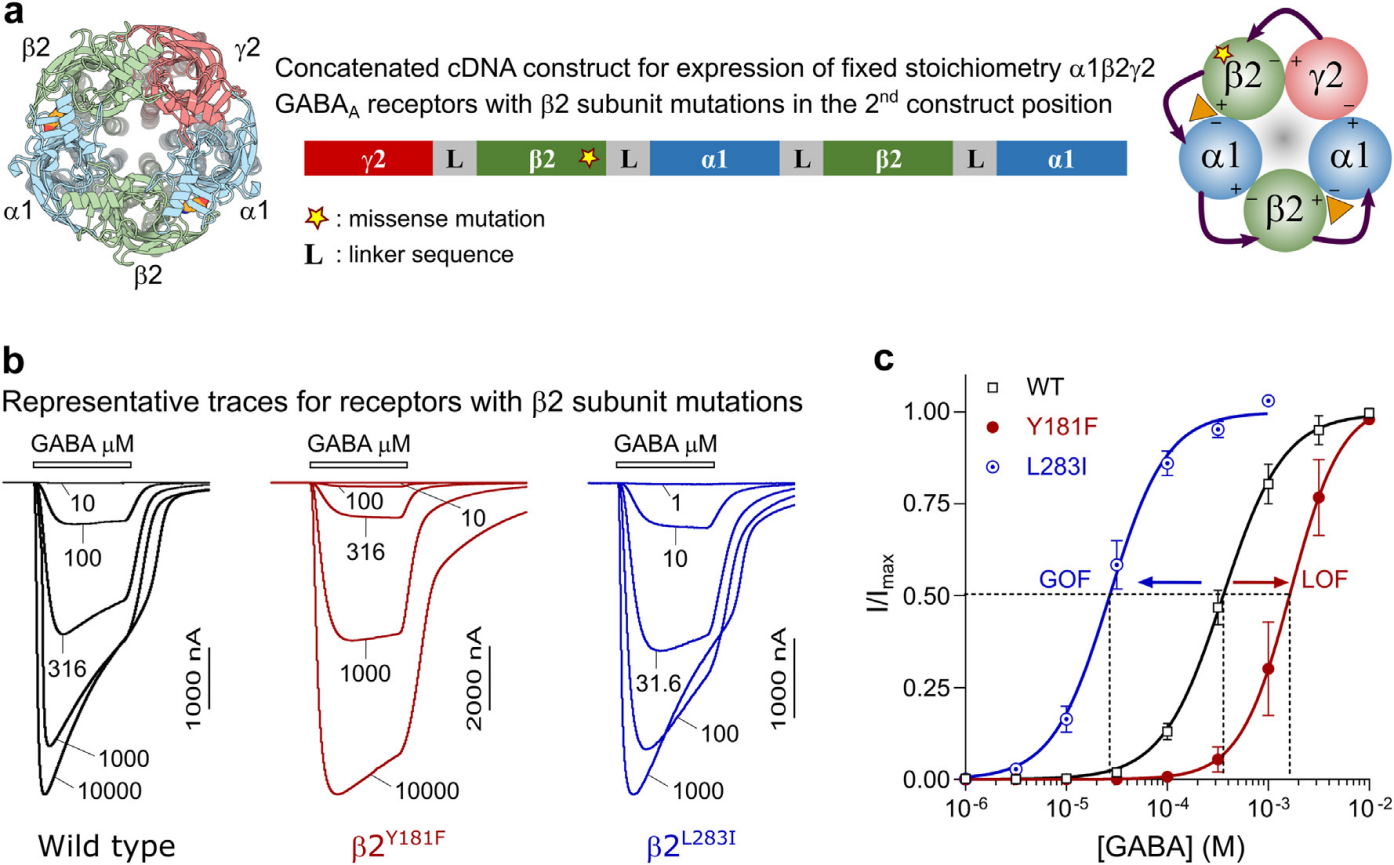
Concatenated construct and representative traces (**a**) Top view of cryo-EM structure of the α1β2γ2 GABA_A_ receptor (6x3z) with bound GABA (left). Pentameric concatenated receptor design for the γ2-β2^star^-α1-β2-α1 cDNA construct utilised to introduce β2 subunit missense mutations (star) in the second construct position only (middle). The five subunits are linked with four linker sequences (L) based on Alanine-Glycine-Serine repeats. The resulting pentameric receptor is portrayed with arrows indicating linkers sequences and the counterclockwise assembly orientation (right). (**b**) Representative electrophysiological traces depicting GABA concentration-response relationships for receptors containing the β2 wildtype (black), β2^Y181F^ (red) or β2^L283I^ (blue) subunit. Bars above the traces depict the 25-s application time with GABA concentrations indicated for each trace in μM. (**c**) GABA concentration-response relationships are depicted as mean ± SD for n = 9–11 biological replicates and the Hill equation was fitted to the data by non-linear regression. The GABA sensitivity is observed from the fitted EC_50_ value and arrows indicate whether mutations cause GOF or LOF in GABA sensitivity.

A pentameric α1β2γ2 receptor contains two β2 subunits (Fig. 1) and since all individuals in this study are heterozygous for their respective *GABRB2* variants, they would be expected to express a mixture of receptor assemblies comprising either zero, one or two variant β2 subunits. Of these, the receptors containing one variant and one wildtype subunit would be expected to constitute the bulk of expressed receptors (50%, assuming a binomial distribution of equal numbers of wildtype and variant subunits) and are therefore the more important combination to investigate. To ensure uniform expression of receptors with one mutant subunit, pentameric concatenated constructs with fixed subunit stoichiometry and arrangement were built for all variants (Fig. 2a). GABA sensitivities as well as total current amplitudes were then systematically assessed for the variant receptors and compared with wildtype receptors using electrophysiology (measured and fitted values as well as statistical comparisons are presented in Supplementary Table S1).

Receptors comprising the β2^Y181F^ and β2^L283I^ mutations represented the functional spectrum observed during the electrophysiological analysis. Wildtype receptors, as well as receptors containing the β2^Y181F^ and β2^L283I^ mutations, responded to GABA in a concentration-dependent manner (Fig. 2b). The receptor sensitivity to GABA was derived by fitting the Hill equation to GABA concentration-response relationships and calculating the concentration that elicits a half maximal receptor response (EC_50_). The β2^Y181F^ and β2^L283I^ mutations significantly altered receptor sensitivity to GABA (Fig. 2c). The β2^Y181F^ mutation caused a 5-fold shift towards lower GABA sensitivity (right-shift), consistent with a LOF trait (ΔLogEC_50_ value = −0.70 ± 0.12, n = 17). Conversely, the β2^L283I^ mutation caused a 13-fold shift toward increased GABA sensitivity (left-shift), consistent with a GOF trait (ΔlogEC_50_ value = 1.10 ± 0.09, n = 15).

**Fig. 3:**
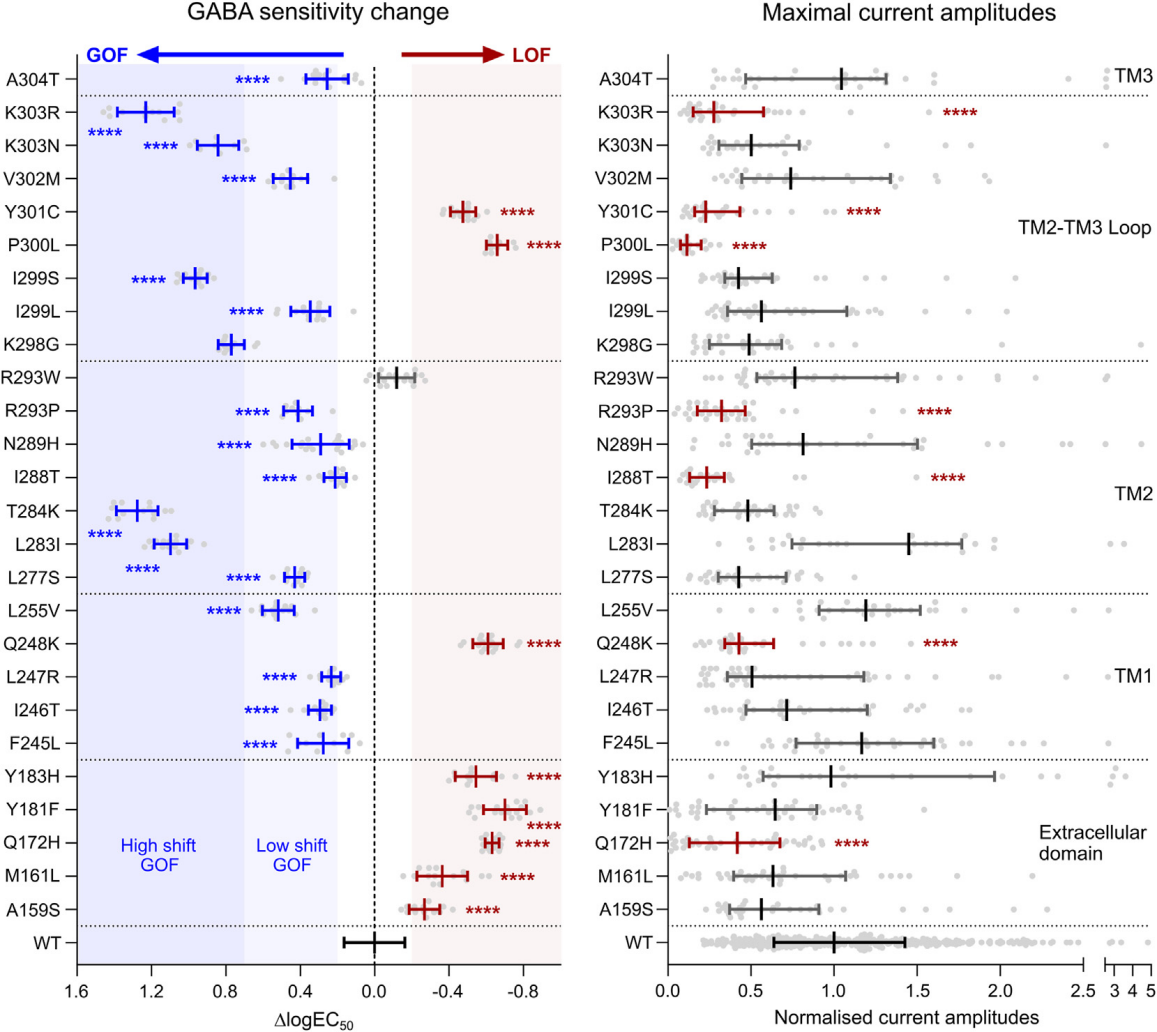
GABA sensitivity changes and maximal GABA-evoked current amplitudes for α1β2γ2 receptors with β2 subunit mutations. (**Left**) Changes in GABA sensitivity between wildtype (WT) and receptors with β2 subunit mutations are presented as mean ΔLogEC_50_ ± SD for n = 222 (WT) or n = 9–22 (β2 mutations) experiments with individual datapoints shown (light grey). Blue indicates mutations that significantly increase GABA sensitivity (GOF), red indicate mutations that significantly reduce GABA sensitivity (LOF) and grey indicate mutations with no significant change. Significance was determined using one-way ANOVA with corrected Dunnetts’ multiple comparisons *post hoc* test (∗∗∗∗, *P* < 0.0001; full detail in the Supplementary table). A change from darker to lighter vertical blue shading at ΔLogEC_50_ = 0.7 guides the separation between High-shift (ΔLogEC_50_ > 0.7) and Low-shift (ΔLogEC_50_ < 0.7) GOF variants. (**Right**) Normalised maximal GABA-evoked current amplitudes are presented as median with IQR for n = 390 (WT) or n = 17–42 (β2 mutations) experiments. Red indicates mutations causing LOF whereas grey indicates mutations with no significant change. Significance was determined using Mann–Whitney U test (∗∗∗∗, *P* < 0.0001; full detail in the Supplementary table). Stars indicate a significantly reduced current amplitude that additionally is below the set threshold 0.5 level (red shading, see methods).

All 26 mutant receptors were functional and exhibited concentration-dependent currents in response to GABA applications. Mean GABA sensitivities were significantly affected by the variants (One-Way ANOVA, *F*(26, 567) = 310; *P* < 0.0001). Eight of the mutations caused LOF by decreasing GABA sensitivity, while 17 mutations caused GOF by increasing GABA sensitivity (Dunnett’s corrected multiple comparison; *P* < 0.0001) (Fig. 3 left). Only the β2^R293W^ mutation did not significantly alter GABA sensitivity. Mutations causing LOF were observed to yield 1.9–5-fold decreases in GABA sensitivity (ΔlogEC_50_ value from −0.27 to −0.70), with mutations causing GOF increasing sensitivity 1.6–19-fold (ΔlogEC_50_ value from 0.21 to 1.28). All five mutations in the extracellular domain led to LOF, while 10 out of 11 mutations in the transmembrane helices led to GOF. Mutations in the M2-M3 loop of the β2 subunit led to either GOF or LOF.

Next, maximal GABA-evoked current amplitudes were evaluated for receptors containing the 26 β2 subunit mutants. A substantial reduction in current amplitude may indicate issues related to either trafficking of receptors to the cell surface or gating efficiency of expressed receptors. Seven of the 26 mutations caused a significant loss of maximal current amplitude with remaining amplitudes varying from 11 to 49% of the wildtype (Mann–Whitney U test; *P* < 0.0001, I_max_ < 0.5) (Fig. 3 right). Four of these mutations, β2^Q172H^, β2^Q248K^, β2^P300L^ and β2^Y301C^, also caused a significant decrease in GABA sensitivity indicating LOF traits for both measured parameters. By contrast, the β2^I288T^, β2^R293P^ and β2^K303R^ mutations caused a significant increase in GABA sensitivity and thus present with a mixed GOF/LOF molecular phenotype. Importantly, the mixed GOF/LOF receptors still retained approximately 33% of the maximal current amplitudes, indicating that none of them are equivalent to a haploinsufficiency scenario. Consequently these variants are kept as part of GOF group in the following and the complexity of the mixed GOF/LOF molecular phenotype is addressed separately.

### ACMG guidelines and cohort segregation

**Supplementary Fig. S1:**
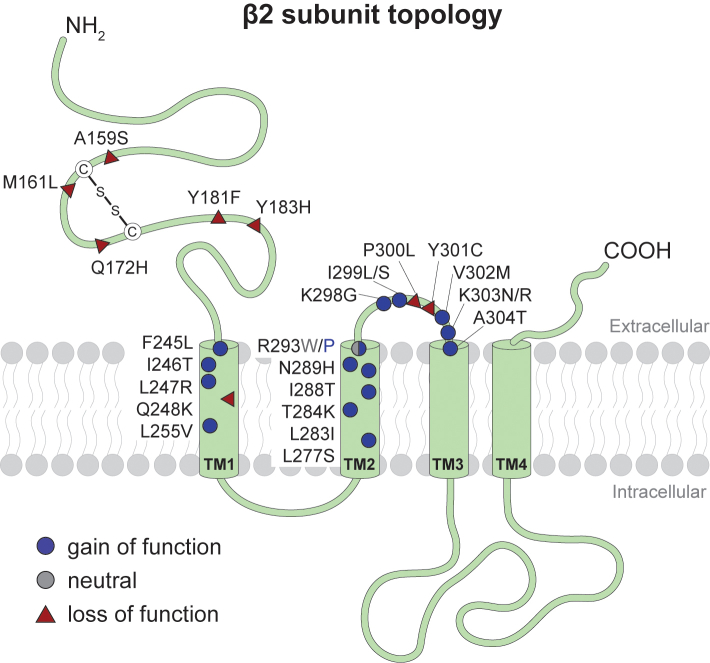
Supplementary Fig. S1. 2-D topology of a GABA_A_ receptor β2 subunit with mapping of GOF and LOF variants.

Functional analysis revealed that 25 *de novo GABRB2* variants caused a significant receptor dysfunction in a well-established functional assay (Supplementary Fig. S1), thus providing strong support for a pathogenic role of these (ACMG guideline codes PS2 and PS3). Only the inherited R293W variant did not cause a significant functional alteration and should therefore be categorised as a variant of uncertain significance (ACMG codes PP1 and BS3). The R293W variant was identified in a family with two affected individuals (daughter and father) who both suffered from intractable epilepsy.^1^ These two individuals were omitted from the phenotypic analysis (clinical information available in Supplementary Table S1). To investigate whether clinical phenotypes correlate with the functional effects of the *GABRB2* variants, the remaining 40 individuals were initially segregated into LOF and GOF sub-cohorts based on the observed changes in GABA sensitivity produced by their respective variants. These two sub-cohorts consist of 13 individuals with LOF variants and 27 individuals with GOF or mixed GOF/LOF variants. The clinical characteristics of focus included age of seizure onset, seizure types, response to treatment, developmental delay/intellectual disability (DD/ID), severe feeding difficulties, hypotonia, microcephaly and movement disorders.

### Phenotypic characterisation of individuals with LOF variants

Thirteen individuals (8 females, 5 males) carried a LOF variant (Table 1, Table 2, and Supplementary Table S1). Age at last follow-up ranged from 3 to 25 years (median = 8.0 years [IQR: 5.0–16]), and no early mortality was reported. All individuals suffered from a neurodevelopmental disorder (NDD) with cognitive impairment from mild (7/13) through moderate (4/13) to severe (2/13) DD/ID. All except one (#7) presented with epilepsy between 5 and 12 months of age (median = 7.5 months [IQR: 6.1–9.0]). Individual #7 was diagnosed with a NDD with severe language impairment, but no epilepsy. Seizures were triggered by fever in 11/12 (92%) of the individuals with epilepsy. The most common seizure types included generalised tonic-clonic seizures (GTCS), focal seizures, atonic seizures, atypical absences, myoclonic seizures and hemiclonic seizures. Electrographic findings varied from normal EEGs (4/11) to EEGs displaying focal/multifocal (6/11) or generalised (3/11) interictal epileptiform discharges (IED). The epilepsy outcome ranged from daily (3/12), monthly seizures (4/12), to yearly seizures (2/12) and seizure freedom (3/12). Epilepsy classification included syndromes within the GEFS+ spectrum (FS+ (2/13), Dravet syndrome/Dravet-like (3/13), unclassifiable fever sensitive DEE (4/13), myoclonic-atonic epilepsy (MAE, 1/13)) and neurodevelopmental disorder with or without epilepsy (3/13).

**Table 1:** Forty individuals with pathogenic loss- or gain-of-function *GABRB2* variants.

#	Sex, age last follow-up	Variant	Variant HGVS	Age at seizure onset	Syndrome	DD/ID	Severe movement disorders	Deceased	Reference
LOF variants									
1	F, 25 y	A159S	p.(Ala159Ser)	12 m	DS-like	Mild ID	No	No	el Achkar et al.^1^ + NI
2	F, 8 y	M161L	p.(Met161Leu)	9 m	FS+	Mild ID	No	No	el Achkar et al.^1^ + NI
3	M, 15 y	Q172H	p.(Gln172His)	9 m	FS+	Mild ID	No	No	Unreported
4	M, 8 y	Y181F	p.(Tyr181Phe)	8 m	DS-like	Mild DD	No	No	el Achkar et al.^1^
5	M, 3 y	Y181F	p.(Tyr181Phe)	6.5 m	DS	Mild ID	No	No	Yang et al.^8^
6	M, 5 y	Y183H	p.(Tyr183His)	6 m	NDD + Epi.	Mild DD	No	No	el Achkar et al.^1^ + NI
7	F, 5 y	Q248K	p.(Gln248Lys)	No	NDD	Mild ID	No	No	Unreported
8	F, 4 y	P300L	p.(Pro300Leu)	5 m	NDD + Epi.	Moderate DD	No	No	el Achkar et al.^1^ + NI
9	F, 6 y	P300L	p.(Pro300Leu)	7 m	DEE	Moderate DD	No	No	Unreported
10	F, 20 y	Y301C	p.(Tyr301Cys)	8 m	DEE	Severe ID	No	No	Unreported
11	F, 15 y	Y301C	p.(Tyr301Cys)	7 m	DEE	Severe ID	No	No	Unreported
12	M, 17 y	Y301C	p.(Tyr301Cys)	6 m	DEE	Moderate ID	No	No	Unreported
13	F, 8.5 y	Y301C	p.(Tyr301Cys)	9 m	MAE	Moderate ID	No	No	Maillard et al.^25^ + NI
GOF variants (Low shift)									
14	F, 10 y	F245L	p.(Phe245Leu)	3 m	NDD + Epi.	ID unspecified	No	No	el Achkar et al.^1^
15	M, 2 y	I246T	p.(Ile246Thr)	5 m	LGS-like	Severe DD	No	2 y: PNA	el Achkar et al.^1^
16	M, 3 y	I246T	p.(Ile246Thr)	2 m	EIDEE	Severe DD	Yes	No	el Achkar et al.^1^
17	M, 10 y	L247R^b^	p.(Leu247Arg)	7 m	DEE	Severe ID	Yes	No	Unreported
18	M, 4 y	L255V	p.(Leu255Val)	5 m	NDD + Epi.	Severe DD	Yes	4 y: SUDEP	el Achkar et al.^1^
19	M, 15 y	L277S	p.(Leu277Ser)	4 y 8 m	DEE	Severe ID	No	No	Hamdan et al.^24^
20	F, 10 y	L277S	p.(Leu277Ser)	2 y	DEE	Severe ID	No	No	Hamdan et al.^24^
21^a^	F, 3 y	I288T^c^	p.(Ile288Thr)	4 m	NDD + Epi.	Moderate DD	Yes	No	el Achkar et al.^1^
22	F, 7 y	N289H	p.(Asn289His)	No	NDD	Moderate ID	No	No	Unreported
23^a^	F, 1.5 y	R293P	p.(Arg293Pro)	No	NDD	Severe DD	Yes	No	Hamdan et al.^24^
24^a^	F, 7 y	R293P	p.(Arg293Pro)	4 y 10 m	NDD + Epi.	Moderate ID	Yes	No	Unreported
25	F, 4 y	I299L	p.(Ile299Leu)	19 m	DEE	Severe ID	Yes	4 y: SE	el Achkar et al.^1^ + NI
26	M, 10 y	V302M	p.(Val302Met)	8 m	NDD + Epi.	Moderate ID	Yes	No	el Achkar et al.^1^
27	F, 17 y	V302M	p.(Val302Met)	6 y	NDD + Epi.	Moderate ID	Yes	No	Unreported
28	M, 9 y	V302M	p.(Val302Met)	No	NDD	Severe ID	Yes	No	Unreported
29	F, 42 y	V302M	p.(Val302Met)	2 y	Rett	Severe ID	No	No	Cogliati et al.^26^
30	M, 14 y	A304T	p.(Ala304Thr)	2 y	NDD + Epi.	Severe ID	No	No	el Achkar et al.^1^ + NI
31	M, 3 y	A304T	p.(Ala304Thr)	1 d	EIDEE	Severe DD	No	No	Unreported
GOF variants (High shift)									
32	M, 2 m	L283I	p.(Leu283Ile)	3 d	EIDEE	Severe DD	NR	2 m: UK	Maillard et al.^25^ + NI
33	M, 17 d	T284K	p.(Thr284Lys)	7 d	EIDEE	Severe DD	NR	17 d: UK	Hamdan et al.^24^
34	M, 6 y	K298G	p.(Lys298Gly)	6 m	EIDEE	DD unspecified	UK	No	Yang et al.^8^
35	M, 3 y	I299S	p.(Ile299Ser)	3 m	IESS	Severe DD	Yes	No	el Achkar et al.^1^
36	M, 1.5 y	K303N	p.(Lys303Asn)	1 d	EIDEE	Severe DD	UK	1.5 y: RSV	Baldridge et al.^27^
37	M, 14 m	K303N	p.(Lys303Asn)	10 d	EIDEE	Severe DD	UK	14 m: PNA	Yang et al.^8^
38^a^	M, 5 y	K303R	p.(Lys303Arg)	3 d	EIDEE	Severe ID	No	No	el Achkar et al.^1^ + NI
39^a^	M, 4 y	K303R	p.(Lys303Arg)	1 d	EIDEE	Severe ID	Yes	No	Hamdan et al.^24^
40^a^	M, 10 y	K303R	p.(Lys303Arg)	1 d	EIMFS	Severe ID	Yes	No	Unreported

Severe movement disorders include dystonia, dyskinesia, hyperkinesia and chorea. Reference indicates whether the information for each individual is: previously unreported, previously reported and updated with with new information (NI) or as previously reported.

DD, developmental delay; DEE, developmental and epileptic encephalopathy; DS, dravet spectrum; EIDEE, early infantile developmental and epileptic encephalopathy; EIMFS, epilepsy of infancy with migrating focal seizures; Epi., epilepsy; FS+, febrile seizures plus; GOF, gain-of-function; IESS, infantile epileptic spasms syndrome; ID, intellectual disability; LGS, Lennox-Gastaut syndrome; LOF, loss-of-function; MAE, myoclonic-atonic epilepsy; NDD, neurodevelopmental delay; PNA, pneumonia; RSV, respiratory syncytial virus; SUDEP, sudden unexpected death in epilepsy; UK, unknown.

a Functional analysis revealed mixed GOF/LOF traits.

b Mosaic (30%).

c Mosaic (degree unknown).

**Table 2:** Summary of phenotypic characteristics of 40 individuals with pathogenic loss- or gain-of-function variants in *GABRB2.*

	LOF (n = 13)	GOF (n = 27)	Low-shift GOF (n = 18)	High-shift GOF (n = 9)
Sex	8 female/5 male	9 female/18 male	9 female/9 male	9 male
Age at last follow-up median (range) [IQR]	8.0 y (3 y–25 y) [5.0–16]	5.0 y (17 d–42 y) [3.0–10]	8.0 y (1.5 y–42 y) [3.0–11]	3.0 y (17 d–10 y) [0.67–5.5]
Epilepsy	12/13	24/27	15/18	9/9
Age of seizure onset median (range) [IQR]	7.5 m (5–12 m) [6.1–9.0]	4.5 m (1 d–72 m) [0.13–23]	8.0 m (1 d–72 m) [4.0–24]	0.10 m (1 d–6 m) [0.033–1.7]
Syndrome	FS+ 2/13DS/DS-like 3/13 MAE 1/13 DEE 4/13 (31%) NDD + epilepsy 2/13 NDD 1/13	EIDEE 8/27 (30%) IESS 1/27 EIMFS 1/27 DEE 5/27 NDD + epilepsy 7/27 (26%) LGS-like 1/27 Rett syndrome 1/27 NDD 3/27	EIDEE 2/18 DEE 4/18 NDD + epilepsy 7/18 (39%) LGS-like 1/18 Rett syndrome 1/18 NDD 3/18	EIDEE 6/9 (67%) IESS 1/9 EIMFS 1/9 DEE 1/9
Seizure triggers	Fever: 11/12 (92%) Photo: 5/12 (42%) Eye rubbing: 1/12 Stress: 1/12 Sono sensibility: 1/12	Fever: 3/22	Fever: 1/14	Fever: 2/8
Seizure outcome	Seizure free: 3/12 Yearly: 2/12 Weekly-monthly: 4/12 (33%) Daily-intractable: 3/12	Seizure free: 6/19 (31%) Yearly: 0/19 Weekly-monthly: 3/19 Daily-intractable: 10/19 (53%)	Seizure free: 5/11 (45%) Yearly: 0/11 Weekly-monthly: 1/11 Daily-intractable: 5/11 (45%)	Seizure free: 1/8 Yearly: 0/8 Weekly-monthly: 2/8 Daily-intractable: 5/8 (63%)
DD/ID	Normal: 0/13 Mild: 7/13 (54%) Moderate: 4/13 (31%) Severe: 2/13	Unspecified: 2/27 Normal/mild: 0/27 Moderate: 5/27 Severe: 20/27 (74%)	Unspecified: 1/18 Normal/mild: 0/18 Moderate: 5/18 (28%) Severe: 12/18 (67%)	Unspecified: 1/9 Normal/mild: 0/9 Moderate: 0/9 Severe: 8/9 (89%)
Language impairment	Normal (full sentences): 3/13 Mild/moderate (phrases): 2/13 Severe (few words): 6/13 (46%) Profound (nonverbal): 2/13	Normal (full sentences): 0/19 Mild/moderate (phrases): 1/19 Severe (few words): 2/19 Profound (nonverbal): 16/19 (84%)	Normal (full sentences): 0/15 Mild/moderate (phrases): 1/15 Severe (few words): 2/15 Profound (nonverbal): 12/15 (80%)	Profound (nonverbal): 4/4 (100%)
Gait	Walking independently: 11/13 (85%) Broad-based/unsteady gait: 2/13 Non-ambulant: 0/13	Walking independently: 3/21 Broad-based/unsteady gait: 8/21 (38%) Non-ambulant: 10/21 (48%)	Walking independently: 2/16 Broad-based/unsteady gait: 8/16 (50%) Non-ambulant: 6/16 (38%)	Walking independently: 1/5 Broad-based/unsteady gait: 0/5 Non-ambulant: 4/5
Dystonia/dyskinesia/hyperkinesia/chorea	0/13	13/22 (59%)	10/18 (56%)	3/4
Neuro-psychiatric/behavioural features	9/11 (82%)	10/23 (43%)	10/18 (56%)	0/5^a^
Feeding difficulties	0/13	7/23 (30%)	1/16	6/7
Hypotonia	5/13 (38%)	15/26 (58%)	9/18 (50%)	6/8 (75%)
Microcephaly	0/13	13/26 (50%)	7/17 (41%)	6/9 (67%)
Early mortality	0/13	7/27 (26%)	3/18	4/9 (44%)

DD, developmental delay; DEE, developmental and epileptic encephalopathy; DS, dravet spectrum; EIDEE, early infantile developmental and epileptic encephalopathy; EIMFS, epilepsy of infancy with migrating focal seizures; FS+, febrile seizures plus; GOF, gain-of-function; IESS, infantile epileptic spasms syndrome; ID, intellectual disability; LGS, Lennox-Gastaut syndrome; LOF, loss-of-function; MAE, myoclonic atonic epilepsy; NDD, neurodevelopmental delay.

a Likely too severe to assess.

Language development ranged from normal (3/13) through mild speech impairment (2/13) to severe/profound language impairment (8/13). All 13 individuals were ambulant although two with a broad-based and unsteady gait. Hypotonia was reported in five out of 13. Otherwise, the neurological examinations were normal, and all 13 individuals had normal head circumference. Four out of 13 had strabismus. The behavioural and psychiatric profile consisted of five individuals with hyperactivity, attention-deficit hyperactivity disorder (ADHD) and/or autistic behaviour with or without severe temper tantrums or aggressive behaviour, one with short attention span and stereotypies and one with mood swings, restlessness and breath holding spells. In addition, individual #10 presented with non-epileptic myoclonus and dyspraxia.

### Phenotypic characterisation of individuals with GOF variants

Twenty-seven individuals (9 females, 18 males) carried a GOF variant (Table 1, Table 2, and Supplementary Table S1). Age at last follow-up ranged from 17 days to 42 years (median = 5.0 years [IQR: 3.0–10]), and seven individuals deceased between the age of 17 days and 4 years (median 14 months [95% CI 0.55–48]). All suffered from a NDD with moderate to severe DD or cognitive impairment from moderate (5/25) to severe/profound ID (20/25), with the degree of DD/ID being unspecified for two individuals. Twenty-four out of 27 suffered from epilepsy with seizure onset between day 1 and 6 years of life (median = 4.5 months [IQR: 0.13–23]). The most common seizure types included focal, tonic, myoclonic and atonic seizures, epileptic spasms and GTCS. A flare-up in seizure frequency during infections and fever was reported in 3/22 (14%). The EEGs showed a variety of abnormalities including focal/multifocal IED, hypsarrhythmia or burst suppression. The background activity was slow in the majority of individuals. The epilepsy severity spanned from daily-intractable seizures (10/19), weekly-monthly (3/19) to seizure freedom (6/19). Information on epilepsy outcome was not available for four individuals and the outcome was “unspecified controlled” in one individual. Epilepsy syndromes included early infantile developmental and epileptic encephalopathy (EIDEE), EIMFS, IESS, DEE, LGS-like or Rett syndrome or a NDD with epilepsy.

Most individuals who were above the age of 2 years at the last follow-up, and for whom data was available, had severe language impairment (18/19) and were either non-ambulant (10/21) or had an unsteady or broad-based gait (8/21). Only one (#14) out of 19 individuals was able to talk in phrases, and only three out of 21 (#14, #21, #34) were reported to have a near to normal gait. Neurological/clinical examinations revealed hypotonia (15/26), hypertonia (2/26), spasticity (6/26), nystagmus (4/26), strabismus (10/24) and ataxia (2/26). Prominent infantile or early childhood onset movement disorders including dystonia, dyskinesia or chorea were observed in 13/22 individuals for whom data was available. The behavioural and psychiatric profile consisted of autism spectrum disorder/autistic features in 5/23, stereotypies in 5/23 and ADHD/hyperactivity, obsessive-compulsive disorder, and anxiety in 2/23 each. Head circumference was reported as normal in 12/26, whereas microcephaly was observed in 13/26 and macrocephaly (#26) was observed in 1/26.

The causes of death for the seven deceased individuals included respiratory failure due to a pneumonia or respiratory syncytial virus (3), sudden unexpected death in epilepsy (SUDEP, 1) and status epilepticus (1). The cause of death is unknown for two individuals.

### Phenotypic features distinguishing LOF and GOF sub-cohorts

**Fig. 4:**
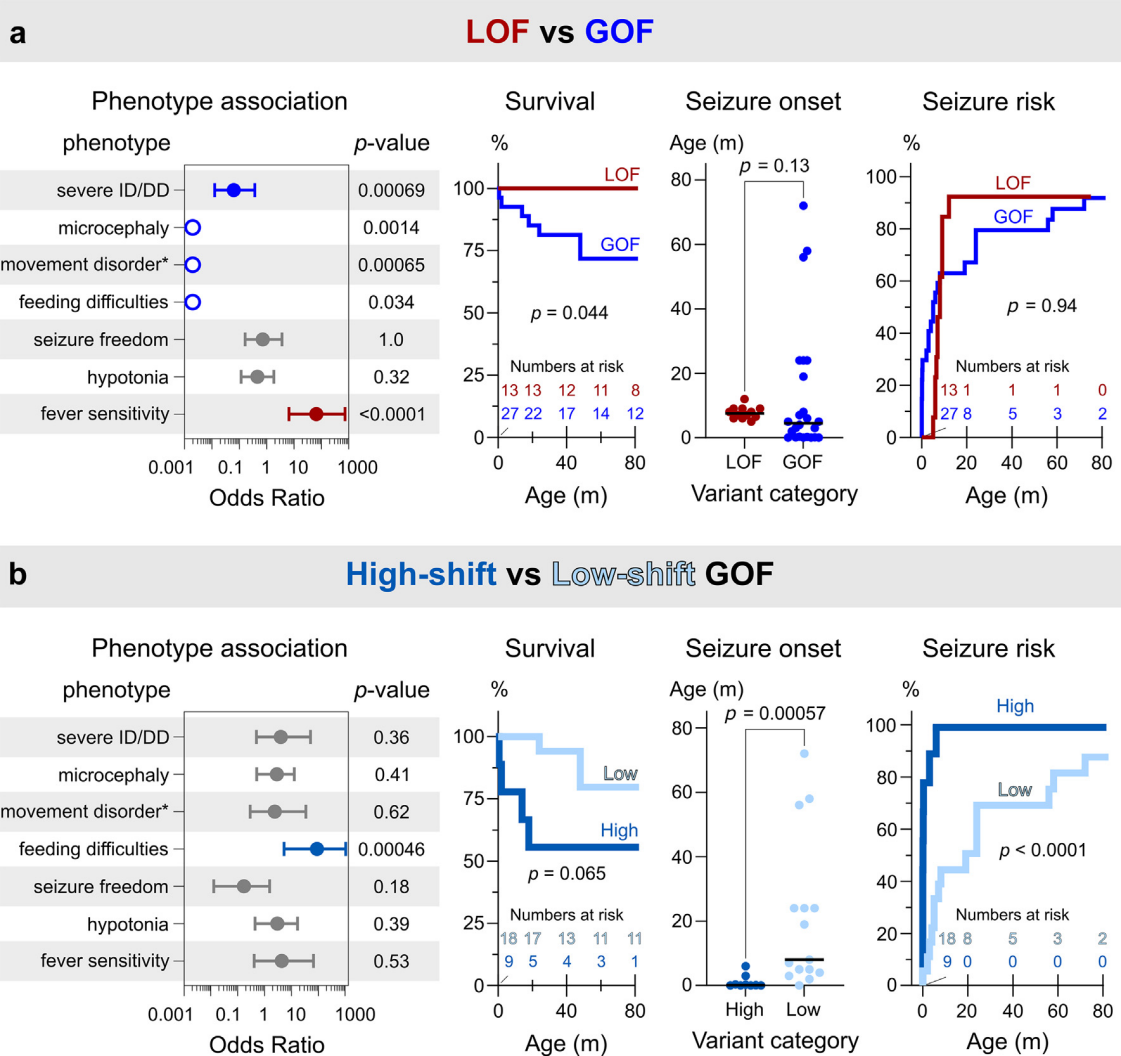
Clinical phenotypes of individuals with LOF and GOF *GABRB2* variants. Selected clinical parameters were assessed for their association with the molecular phenotype of the respective variants. (**a**) Comparison of LOF (n = 13) vs GOF (n = 27) sub-cohorts. (**b**) Comparison of High-shift GOF (n = 9) vs Low-shift GOF (n = 18) groups. Odds ratio (OR) analyses of phenotype–genotype associations are presented with the centre circle denoting the OR and 95% confidence interval. Blue indicates significant enrichment in individuals with GOF (a) or High-shift GOF (b), red significant enrichment in individuals with LOF and grey no significant difference between compared individuals. Open circles without confidence intervals indicate data where one category contains 0 or 100% of individuals and the OR and CI cannot be determined. Statistical analyses were performed using two-sided Fisher’s exact test resulting in the indicated *P* values. Statistics for survival analyses, seizure onset and seizure risk were performed using Mantel–Cox, Mann–Whitney and Mantel–Cox tests, respectively, with the obtained *P* values indicated. Number of individuals at risk in survival and seizure risk analyses are indicated at five timepoints, “m” refers to months in age of seizure onset and “movement disorder∗” refers to dystonia, dyskinesia, hyperkinesia, or chorea.

To identify key phenotypic features differentiating individuals with LOF and GOF variants, prominent clinical features (Table 2) were formally compared (Fig. 4a). Individuals with LOF variants exhibited a lower prevalence of severe DD/ID (15% LOF vs 74% GOF; Odds Ratio (OR) = 0.063 [95% CI: 0.013–0.37]; *P* = 0.00069, Fisher’s Exact Test), microcephaly (0% vs 50%; OR ND; *P* = 0.0014), prominent movement disorders such as dystonia, dyskinesia and/or chorea (0% vs 59%; OR ND; *P* = 0.00065) and severe feeding difficulties (0% vs 30%; OR ND; *P* = 0.034) compared to individuals with a GOF variant. In contrast, individuals with a LOF variants were associated with a greater prevalence of fever sensitivity (92% vs 14%; OR = 66 [6.5–730]; *P* < 0.0001). Despite these clear differentiating factors, no marked variation was noted between individuals with LOF or GOF variants regarding the prevalence of seizure freedom (25% vs 31%; OR = 0.72 [0.17–3.6]; *P* = 1.0) or hypotonia (38% vs 58%; OR = 0.46 [0.12–1.9]; *P* = 0.32). However, a survival analysis revealed that individuals harbouring LOF variants had lower prevalence of early mortality (0% vs 26%; Mantel-Haenszel Hazard Ratio (HR) = 0.20 [95% CI: 0.043–0.95]; *P* = 0.044, Mantel–Cox test) (Fig. 4a).

The median age of seizure onset was similar between individuals harbouring a LOF or a GOF variant (7.5 months [IQR: 6.1–9.0] vs 4.5 months [IQR: 0.13–23], respectively; *P* = 0.13, Mann Whitney test) (Fig. 4a). Intriguingly, a markedly higher variance in the age of onset was found for the GOF compared to the LOF sub-cohort (F-test, *F*_(23,11)_ = 120; *P* = 8.0 × 10^−9^). Furthermore, three individuals with NDD and ID never developed seizures in the GOF sub-cohort underscoring the wide phenotypic spectrum within the GOF sub-cohort itself. An incidence plot suggested that the spectrum of age of seizure onset for GOF variants could be the result of two different groups of individuals: one with very early age of onset (three months or less) and another with later age of onset.

### Mixed GOF/LOF variants

To investigate whether different biophysical properties of the variants contribute to the wide phenotypic variability within the GOF sub-cohort, the impact of significant changes in maximum current amplitudes was assessed in conjunction with the results from the GABA sensitivity analysis (Fig. 3). Within the GOF sub-cohort, 21 individuals carried 14 distinct variants that did not exhibit a significant reduction in maximal current amplitudes. However, six individuals harboured the I288T, R293P and K303R variants, that in addition to increased GABA sensitivities displayed significantly reduced current amplitudes (Table 1 and Supplementary Table S1). The loss of maximal current amplitude in these cases might signify a mixed GOF/LOF molecular phenotype, whereby reduced surface expression and/or gating efficiency could diminish the relevance of changes in GABA sensitivity. Depending on the degree of current loss, this could be inconsequential or lead to an intermediate clinical phenotype, possibly resembling those observed in the LOF sub-cohort.

Among the six individuals with GOF/LOF variants, one did not have epilepsy, while the remaining developed epilepsy between 1 day and 58 months of age (median = 0.10 months [IQR: 0.033–31]). No differences in age of seizure onset between individuals with LOF, GOF only or GOF/LOF variants were identified (Kruskal–Wallis test; *P* = 0.15). Next, clinical phenotypes for individuals with GOF/LOF variants were compared with the LOF sub-cohort. Individuals with GOF/LOF variants exhibited a greater prevalence of severe DD/ID (67% (4/6) GOF/LOF vs 15% LOF; OR = 13 [95% CI: 1.3–90]; *P* = 0.031, Fisher’s Exact Test), microcephaly (67% (4/6) vs 0%; OR ND; *P* = 0.0049), movement disorders such as dystonia, dyskinesia and/or chorea (83% (5/6) vs 0% OR ND; *P* = 0.00070) and severe feeding difficulties (50% (3/6) vs 0%; OR ND; *P* = 0.042) compared to individuals with LOF variants. In contrast, individuals with GOF/LOF variants had a lower prevalence of fever sensitivity than those with LOF variants (0% (0/5) vs 92%; OR ND; *P* = 0.0010). There were no differences in the prevalence of seizure freedom (50% (2/4) vs 25%; ORs 3.0 [0.32–24]; *P* = 0.55) or hypotonia (50% (2/4) vs 38% (5/13); 0.77 [0.12–6.3]; *P* = 1.0).

Despite the low numbers of individuals, there was thus no evidence that a GOF/LOF molecular phenotype leads to clinical phenotypes resembling those associated with LOF variants for these three specific variants. For all the clinical features analysed, individuals with GOF/LOF variants exhibited similarities to those with GOF-only variants. Hence, it is unlikely that GOF/LOF account for the phenotypic variance within the GOF sub-cohort. This rules out the possibility that individuals with a GOF/LOF variant have been misallocated or form a distinct group within the GOF variants.

### High-shift GOF vs low-shift GOF

As the measure of GABA sensitivity used to define GOF is quantifiable rather than binary, it was next assessed whether the substantial ∼10-fold divergence in the magnitudes of GABA-sensitivity increase correlates with the wide phenotypic spectrum of GOF *GABRB2* variants. For this analysis, individuals with GOF variants were divided into two groups: (i) High-shift GOF, comprising variants with GABA sensitivity increases above 5-fold (ΔlogEC_50_ value > 0.7); and (ii) Low-shift GOF, comprising variants exhibiting GABA sensitivity increases up to 5-fold (ΔlogEC_50_ value < 0.70). Notably, a 5-fold change approximates the midpoint of the observed sensitivity spectrum (Fig. 3).

Nine individuals carried a High-shift GOF variant characterised by 5.9–19-fold increases in GABA sensitivity (ΔlogEC_50_ 0.77–1.28), while 18 individuals harboured a Low-shift GOF variant with 1.6–3.3-fold increases in GABA sensitivity (ΔlogEC_50_ 0.21–0.52). Individuals with High-shift variants experienced an earlier median age of seizure onset (0.10 months [IQR: 0.033–1.7]) compared to those with Low-shift variants (8.0 months [IQR: 4.0–24]; *P* = 0.00057, Mann–Whitney test). An incidence plot further confirmed the differences between these groups (HR = 25 [95% CI: 6.0–100]; *P* < 0.0001) (Fig. 4b). The reported epilepsy syndromes also varied between the two groups. In the High-shift group, diagnoses included early onset DEEs such as EIDEE (6/9), IESS (1/9) or EIMFS (1/9). Conversely, individuals in the Low-shift group were diagnosed with DEE (EIDEE (2/18), unclassified (4/18), LGS-like (1/18)), Rett syndrome (1/18) neurodevelopmental disorders with epilepsy (7/18) and without epilepsy (3/18). Furthermore, EEG abnormalities were more frequently reported in the High-shift group, including hypsarrhythmia (38% (3/8) High-shift vs 7% (1/14) Low-shift) and burst suppression (50% (4/8) vs 7% (1/14)).

Individuals in the High-shift group exhibited a higher prevalence of severe feeding difficulties (86% vs 6%; OR = 90 [5.4–1100]; *P* = 0.00046, Fisher’s Exact Test) (Fig. 4b). However, no differences were observed in the prevalence of other comorbidities including severe DD/ID (89% vs 67%; OR = 4 [0.50–51]; *P* = 0.36), microcephaly (67% vs 41%; OR = 2.9 [0.51–13]; *P* = 0.41), movement disorders (75% vs 56%; OR = 2.4 [0.29–35]; *P* = 0.62), seizure freedom (13% vs 45%; OR = 0.17 [0.013–1.6]; *P* = 0.18), hypotonia (75% vs 50%; OR = 3 [0.45–17]; *P* = 0.39), or fever sensitivity (25% vs 7%; 4.3 [0.41–67]; *P* = 0.53) between the High-shift and Low-shift GOF groups. Although no significant difference in early mortality was observed (HR = 4.9 [0.9–27]; *P* < 0.067), the limited number of individuals made drawing a firm conclusion difficult.

Overall, it is clear that the magnitude of change in GABA sensitivity correlates with the age of seizure onset, and greater changes in the ΔlogEC_50_ values were also associated with different reported epilepsy syndromes, EEG abnormalities and greater prevalence of severe feeding difficulties. Thus, the span in the absolute magnitude of GABA sensitivity changes likely contributes to the large variance in severity within the GOF sub-cohort, which may also be confounded by earlier seizures increasing the severity of the disorder in the High-shift group.

### Receptor desensitisation

**Fig. 5:**
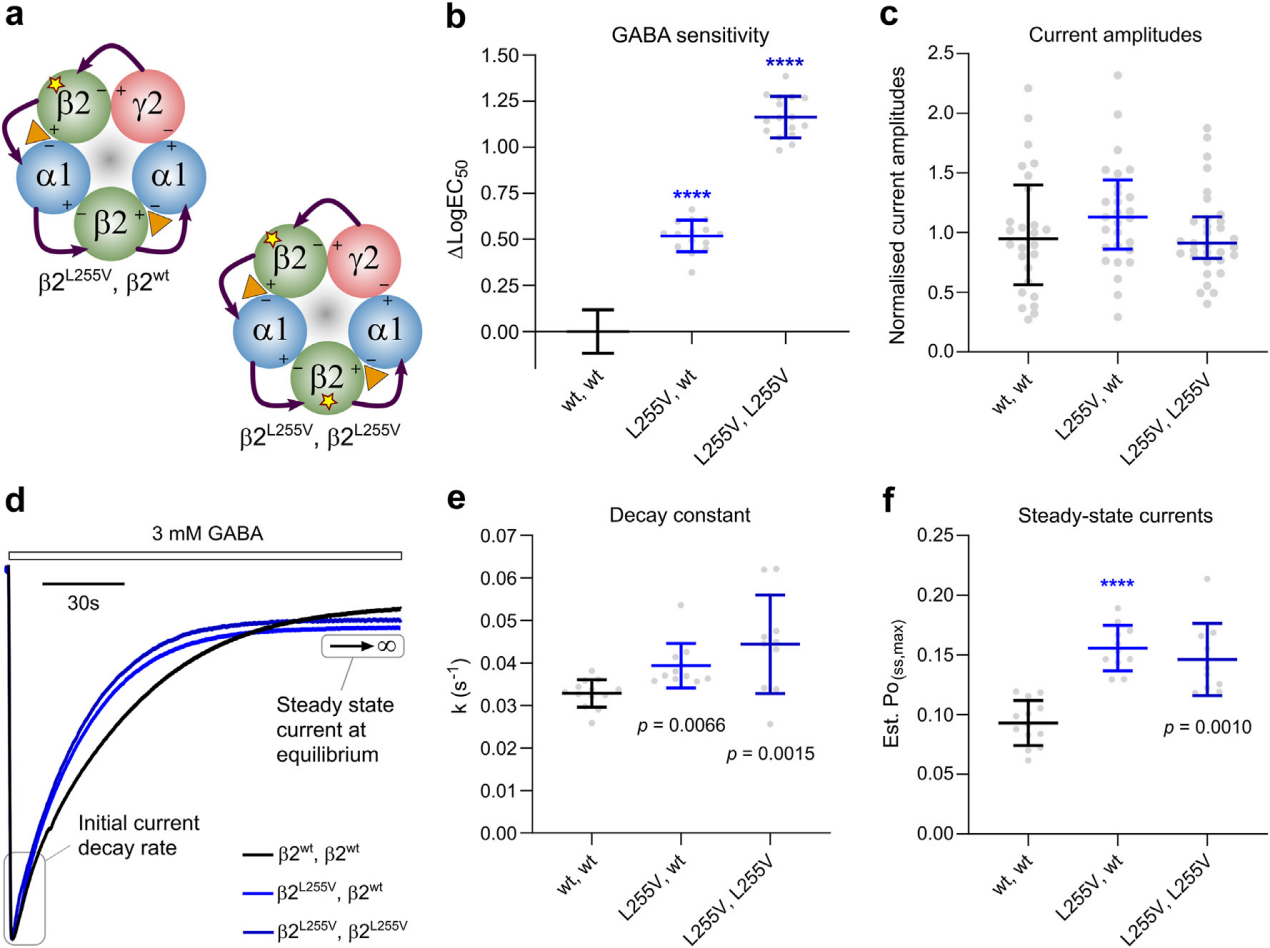
GABA sensitivity changes, maximal current amplitudes, and desensitisation properties for β2^L255V^-containing receptors. (**a**) Two pentameric concatenated constructs were designed with β2^L255V^ mutations (star) to reflect a heterozygous patient condition where receptors can have a single (β2^L255V^, β2^wt^) or two (β2^L255V^, β2^L255V^) variant subunits. (**b**) Changes in GABA sensitivity for the indicated receptor types are presented as mean ΔLogEC_50_ ± SD for n = 15–17 experiments and statistical analysis was performed using one-way ANOVA with corrected Dunnetts’ post-hoc test values depicted in the panel (∗∗∗∗, *P* < 0.0001). (**c**) Normalised maximal GABA-evoked current amplitudes are depicted as median with IQR for n = 26–33 experiments for the indicated receptor types. Statistical analysis was determined using Mann–Whitney U test and no significant differences were observed (Supplementary table). (**d**) Representative traces of responses to 150-s applications of 3 mM GABA at β2^wt^, β2^wt^ (black), β2^L255^, β2^wt^ (blue) and β2^L255V^, β2^L255V^ (dark blue) receptors for illustration of current decay rates and steady-state current amplitudes. Traces were fitted to an exponential decay function to assess the initial current decay rate constant (k) and the estimated open probability at equilibrium (Est. P_O_ (_SS, max_)). (**e, f**) Current decay rates (e) and steady-state current amplitudes at equilibrium (f) are presented for the indicated receptor types as mean ± SD for n = 10–13 experiments. Statistical analysis was performed using a non-parametric one-way ANOVA Kruskal–Wallis test with Dunn’s *post hoc* test values depicted in the panels (∗∗∗∗, *P* < 0.0001).

Intriguingly, some individuals carrying *GABRB2* GOF variants located in the M1 helix exhibited severe phenotypes despite these variants yielding low magnitude shifts in GABA sensitivity. Recently, we reported that more severe phenotypes observed in individuals with *GABRB3* GOF variants in M1 could be attributed to decreased receptor desensitisation.^36^ In the *GABRB2* Low-shift GOF group, one individual (#18) harboured the L255V variant, which is a paralogue of the *GABRB3* L256Q variant previously shown to cause decreased desensitisation. This individual suffered from treatment-resistant epilepsy with severe movement disorders and moderate developmental delay, ultimately succumbing to SUDEP at the age of 4 years. To investigate whether the L255V variant also affects desensitisation properties, we created an additional concatenated construct with two mutated β2^L255V^ subunits (Fig. 5a). Single- and double-mutant receptors were then assessed for GABA sensitivities, maximal current amplitudes, and desensitisation properties. Desensitisation parameters evaluated included rate of current decay (k) and estimated steady-state current at equilibrium (Est. P_O(ss,max)_).

Receptors containing a mutated β2^L255V^ subunit exhibited distinct GABA sensitivity compared to the wildtype receptor (one-way ANOVA (*F*_(2, 45)_ = 460; *P* < 0.0001). The double-mutant receptor showed a 14-fold increase in GABA sensitivity, while the single-mutant receptor had a 3.3-fold increase (Fig. 5b). There were no changes in the maximal GABA-evoked current amplitudes (Fig. 5c). Both β2^L255V^-containing receptors displayed altered current decay rates (Kruskal–Wallis statistic = 14; *P* = 0.00093), albeit the increases were of modest 20–35% magnitude (Fig. 5d and e). Additionally, the mutant receptors altered steady-state currents at equilibrium (Kruskal–Wallis statistic = 23; *P* < 0.0001) with larger increases of 57–67% compared with the wildtype (Fig. 5f). These observations suggest that the L255V variant has additive GOF effects on GABA sensitivity as more variant subunits are introduced in the receptor complex. Furthermore, the variant receptors exhibited decreased desensitisation properties, similar to observations for the paralogous *GABRB3* L256Q variant.

## Discussion

In the present study, we collected a cohort of 42 individuals with presumed pathogenic missense variants in the *GABRB2* gene. Affected individuals harboured 26 different heterozygous missense variants and displayed a spectrum of neurodevelopmental disorders. Functional assessment of the variants demonstrated marked GABA_A_ receptor dysfunction for 25 of the 26 variants and both GOF and LOF alterations were observed. Only the R293W variant found in a daughter and father with intractable epilepsy did not significantly alter the functional parameters analysed. The 25 variants with functional implications all occurred *de novo* or presumed *de novo* in 40 affected individuals. Genotype-phenotype correlation analysis revealed that individuals with *GABRB2* missense variants generally segregate into a GOF and LOF sub-cohorts with distinct clinical characteristics.

### Key clinical predictors for GOF and LOF variants

**Fig. 6:**
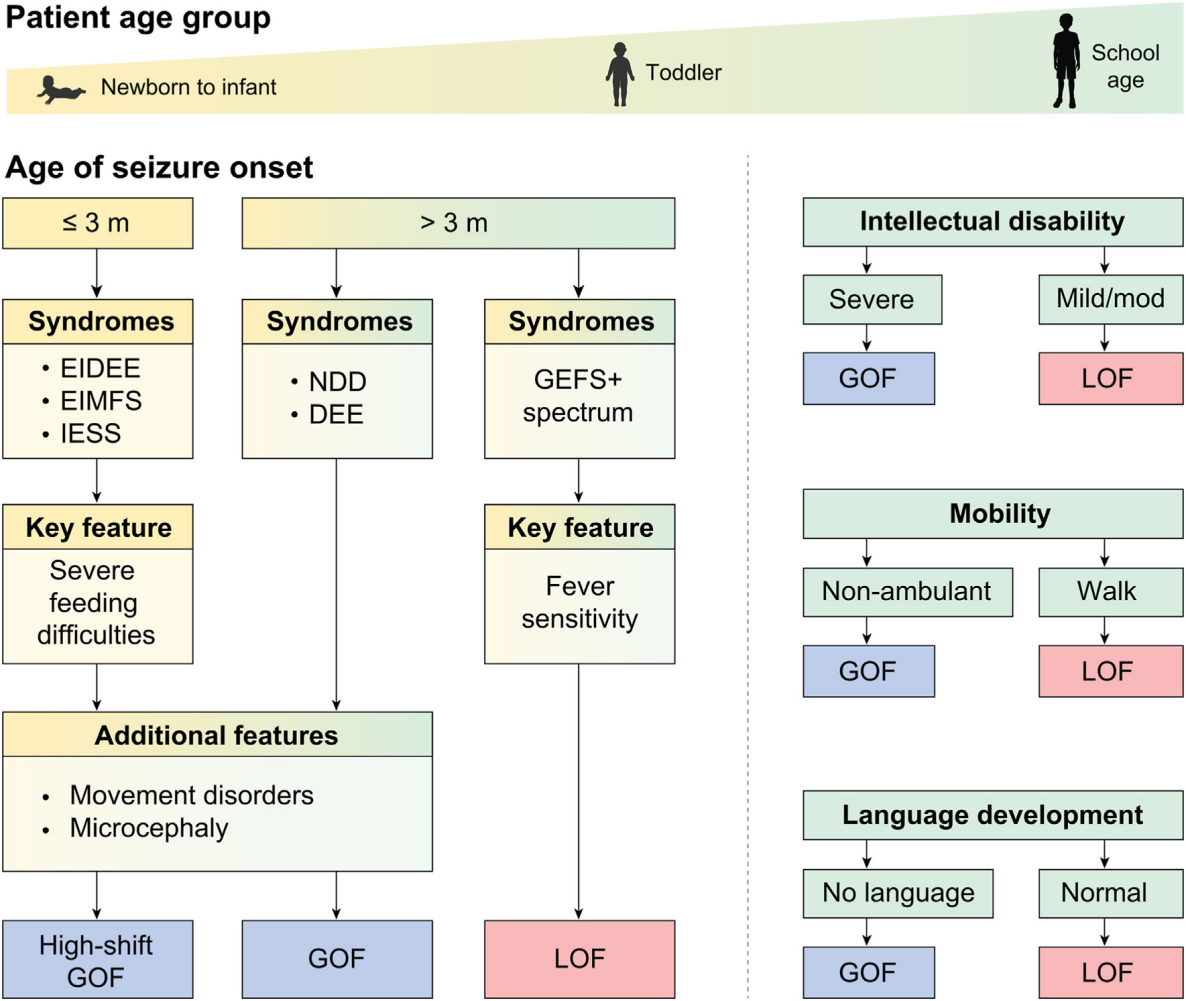
Clinical indicators for GOF and LOF phenotypes. In cases where functional analysis is not available, clinical biomarkers may be used to predict whether a newly identified variant causes GOF or LOF. Age of seizure onset is a strong indicator of GOF disease in cases of early infantile onset and is associated with variants causing High-shift GOF. Severe movement disorders and microcephaly are strongly associated with GOF disease while epilepsy syndromes in the GEFS+ spectrum with fever sensitivity are strongly associated with LOF disease. As the child ages, intellectual disability, independent mobility, and language development support the early indicators. For cases that are not clearly defined by these indicators, functional analysis is needed to determine the effects of the variant.

Understanding whether a pathogenic variant in a gene leads to an overactive (GOF) or underactive (LOF) encoded protein is a prerequisite to facilitate improved clinical outcomes in terms of diagnosis, counselling, and ideally also treatment. Functional analysis is, however, relatively slow, and not always possible. Therefore, clear clinical indicators from the established cases can be utilised to determine the likely functional category of newly identified variants and aid in predicting the progression of the disorder in the affected individual (Fig. 6).

This study identified a key clinical predictor of very early age of seizure onset (three months or less) to be exclusive for GOF variants. Individuals that presented with seizures at these young ages with EIDEE, EIMFS or IESS syndromes were likely to have a large change in GABA sensitivity associated with the most severe High-shift GOF variants. Similarly, severe feeding difficulties were exclusive to individuals with GOF and predominantly observed in individuals carrying High-shift GOF variants.

When individuals presented with seizures at greater than 3 months of age, the variability in the age of onset in the GOF sub-cohort prevents this measure from being used to distinguish between the GOF and LOF. Nevertheless, other key indicators found exclusively for individuals with GOF variants were microcephaly (50%) and the presence of movement disorders generally regarded as basal ganglia dysfunction, including dystonia, dyskinesia, hyperkinesia, and chorea (59%). Such movement disorders have previously been reported in individuals with *GABRB2* variants,^1^ and we demonstrate that they are strongly linked to GOF-associated disorders.

Finally, syndrome classifications differed between the GOF and LOF sub-cohorts. Individuals harbouring a GOF variant were more likely to present with severe forms of DEEs including EIDEE, EIMFS and IESS. In contrast, almost all individuals with LOF variants presented with seizures in the age of 5–12 months and fever sensitivity was near universal (92%). Individuals with LOF variants typically presented with syndromes within the GEFS+ spectrum including FS+, Dravet or Dravet-like phenotypes, MAE or unclassifiable DEEs with fever sensitivity (Fig. 6).

### Clinical features that emerge with age

As the individual matures, other substantive differences emerge between the GOF and LOF sub-cohorts. Although individuals in both sub-cohorts were affected, the impact of GOF variants on cognitive and global development was more pronounced than LOF variants. Severe DD/ID was highly prevalent (74%) for individuals with GOF, whereas most individuals with LOF variants developed mild/moderate (85%) ID. Similarly, while language development was impaired for individuals with LOF variants with less than half (39%) obtaining more complex communication skills, the extent of impairment was greater for GOF with the majority (84%) of individuals reported as non-verbal. Furthermore, all individuals with LOF learned to walk independently, although several with an unsteady gait, whereas half of the individuals with GOF variants were reported to be non-ambulant (Fig. 6).

The response to anti-seizure treatment was not markedly different between the GOF and LOF sub-cohorts with seizure freedom achieved in approximately 30% of individuals in either sub-cohort. However, only one individual in the High-shift GOF group achieved seizure freedom indicating that these individuals are more difficult to treat. Early mortality was absent in the LOF sub-cohort but reported in approximately a quarter of the GOF sub-cohort. Notably, two individuals with an age of onset of five and eighteen months with Low-shift GOF variants died of SUDEP and status epilepticus, demonstrating that the risk of early mortality is not restricted to individuals presenting with neonatal onset seizures.

Finally, while individuals in the GOF and LOF sub-cohorts generally follow these disease progressions, there is a small group of individuals for which the phenotypes are difficult to differentiate. Five individuals with GOF variants presented with a phenotype that did not include any of the clear predictors. These individuals had moderate to severe ID, were ambulant and one was able to talk in phrases while another could say a few words. Apart from the observation that only one of the five had fever sensitivity, the phenotype of these individuals was indistinguishable from the more severe end of the LOF spectrum. Hence, functional assessment would be required to classify these individuals.

### What do the mixed GOF/LOF variants overall resemble?

Until very recently, loss of maximal GABA-evoked current amplitudes has been used as a primary parameter to classify *GABR* variants as LOF.^12^ A model where loss of surface expression or gating deficits determines the phenotype might have intuitive appeal; after all, if there are no active receptors at the neuronal cell surface any other changes measured in an *in vitro* model would be irrelevant to the phenotype. On the other hand, it is inherently difficult to assess whether a loss of receptor current observed in a heterologous expression system translates into actual changes in neuronal synapses.^12^ In this study, three variants were identified that significantly increased GABA sensitivities but also significantly reduced maximum current amplitudes in the functional assay. While only harboured by six individuals, these I288T, R293P and K303R variants with mixed GOF/LOF molecular characteristics allowed a preliminary interrogation of the relative importance of changes in GABA sensitivity vs changes in maximal current amplitudes to the clinical phenotype. Three of the six individuals presented with seizures within the first 3 days of life, five presented with chorea or dystonia, four with microcephaly and none with fever sensitivity. Using the clinical predictors outlined above (Fig. 6), all six individuals with a GOF/LOF variant would thus be predicted to harbour a GOF variant. Hence, the clinical phenotypes demonstrate that changes in GABA sensitivity represent the key major driver for the overall outcome for the three specific GOF/LOF variants. This underscores that erroneous conclusions might be reached from assuming that statistically significant loss of current amplitude *in vitro* necessarily translates into loss of receptor density in synaptic spaces. Approximately 33% current amplitude remained for the GOF/LOF variants measures in this study, suggesting that current reductions markedly greater than the ∼70% are required for this parameter to define the clinical phenotype for *GABRB2*.

### How do individuals with variants *GABRB2* compare with other *GABR* genes?

The data presented here adds *GABRB2* to the list of *GABR* genes for which both GOF and LOF variants have been identified and characterised, thus further establishing GOF variants as a common phenomenon in *GABR* genes.^9^^,^^11^^,^^13^^,^^21^, ^22^, ^23^ Moreover, as 17 out of the 20 transmembrane domain *GABRB2* variants caused GOF, the functional data presented here corroborates previous observations that variants in the transmembrane domains of GABA_A_ receptor subunits have a high likelihood of causing GOF.^11^ Interestingly, several of these *GABRB2* GOF variants are paralogs of previously described *GABRB3* variants suggesting that paralog variants often lead to the same functional outcome within the same subunit class. The phenotypic spectrum of individuals harbouring *GABRB2* variants observed here is in many ways comparable to observations in the recent reports for *GABRA1*,^13^ *GABRB3*^11^ and *GABRD*,^22^ and in all cases the spectrum correlates with GOF and LOF categories based on the functional change in GABA sensitivity.

Given that the GOF sub-cohorts in this study and the *GABRB3* study^11^ are of similar size with 26 and 29 individuals, respectively, it is obvious to compare these. While different GABA_A_ receptor subtypes differ in their spatial and temporal distributions in the brain, severe DD/ID is almost ubiquitous in individuals with *GABRB2* and *GABRB3* GOF variants, as is fever sensitivity in individuals with LOF variants. Additionally, microcephaly is a prominent feature for individuals harbouring GOF variants in either of the two genes. Intriguingly, early-onset DEEs were frequently observed for both individuals with *GABRB2* and *GABRB3* GOF, but while the peculiar epilepsy syndrome EIMFS was more prevalent in the *GABRB3* GOF sub-cohort, EIDEE with a burst suppression pattern occurred at a higher frequency in the *GABRB2* GOF group. Despite the similarities, there are also notable divergences. While also present in individuals with *GABRB3* GOF, the high prevalence of movement disorders generally considered to depend on basal ganglia dysfunction (i.e., dystonia, chorea, dyskinesia and athetosis) is striking in individuals with *GABRB2.* This could suggest that β2-containing receptors play a relatively greater role in the disinhibitory circuits in the basal ganglia that initiate movement.

Interestingly, four individuals carrying the same *GABRB2* LOF variant, Y301C, all suffered from reflex seizures (eyelid myoclonia or myoclonic seizures) triggered by light, photostimulation, stress, sound, or eye rubbing. In one individual (#12) the photosensitivity was so prominent that it required the use of shutters, sunglasses, and darkness in the home. The paralogous *GABRB3* LOF variant Y302C has been reported in four individuals with either focal epilepsy or intractable DEEs including IESS and mild to severe intellectual disability, yet none of these individuals were reported to have reflex seizures, and only one out of four were fever sensitive.^11^^,^^28^^,^^38^ Further studies are warranted to elucidate if reflex seizures are a valid predictor of *GABRB2* LOF disease or whether this phenomenon is specifically linked to this recurrent variant.

To date, five individuals have been described with GOF variants in *GABRA1* encoding the α1 subunit.^13^ Intriguingly, only three of these presented with epilepsy yet all presented with NDD and ID. Due to limited cohort size of individuals with *GABRA1* GOF variants, further studies are warranted to elucidate the phenotypic similarities and differences between *GABRB2* and *GABRA1* GOF disease. Given that the distribution of α subunit expression is typically more localised than β subunit expression, it is reasonable to speculate that individuals with variants across the various α subunits may show greater divergences in clinical phenotypes than observed for the β subunits and that these phenotypes will be more associated with the specific brain region where the respective α subunits are expressed.

### Limitations

There are several limitations to this study. Inherent to research on rare genetic disorders, the number of affected individuals available is limited. As a result, sparse-data bias may occur in situations including where odds-ratio estimates exhibit unrealistically large confidence intervals for rare indications or remain indeterminable for indications with complete penetrance in one group. This limitation is particularly pronounced for the LOF group, since variants in the M1-M3 transmembrane helixes were prioritised to ensure a higher representation of individuals carrying GOF variants. Next, the geographic diversity of individuals, reliance on clinical data from literature, retrospective information obtained from treating physicians or clinical geneticists, and variations in drug treatment regimens introduce potential confounding factors and may contribute to dataset heterogeneity. Consequently, these limitations hinder comprehensive phenotypic descriptions, particularly for rare indications, and limit our ability to assess the impact of age, ethnicity, treatment course, confounding variables, and other factors on the observed phenotypes. Finally, there are also limitations in implementation of these finding into clinical practice. Functional studies, although informative, are time-consuming and not always feasible in a diagnostic setting. Even when functional studies are conducted, a diagnosis of GOF or LOF may not significantly alter clinical care for the majority of individuals. Addressing these limitations should be a focal point for future research.

### Conclusions

In summary, the data presented here demonstrates that genetic variants in the *GABRB2* gene may cause GOF as well as LOF and that this divergence correlates with disease manifestations. Specifically, severe forms of DEE and movement disorders were associated with GOF variants, whereas milder forms of neurodevelopmental disorders and epilepsies within the GEFS+ spectrum were associated with LOF variants. The observation that greater shifts in GABA sensitivity are associated with more severe disease represents an important advancement in the understanding of GABA_A_ receptor associated DEEs. The clinical biomarkers described here will enhance diagnostic accuracy and aid future clinical trials for individuals with *GABRB2* disease. Given that GOF GABA_A_ receptor disease has only recently been recognised, current treatment options are inadequately tailored to address this specific type of receptor malfunction. Therefore, there is an urgent need for future drug development and treatment strategies specifically targeting GOF disease.

## Contributors

PKA, MC and RSM conceptualised the study, acquired funding and managed resources. NAM, PKA and VWYL designed and collected data for the functional studies (generated cDNA constructs and performed electrophysiological experiments). NAM and PKA analysed and interpreted functional data (electrophysiological experiments). NAM, PKA, VWYL, HHC, SOR, PC, GR, MC, AAJ, NLA and RSM interpreted and curated data (genotype–phenotype correlation analysis). KMJ, YMY, AVD, CM, ALB, AR, CP, JK, PB, SM, MT, NS, DA, SA, SB, PM, MM, VS, RM, JRL and SW recruited and phenotyped participants. NAM, PKA, HCC, NLA, and RSM conducted literature search, produced figures, and wrote the manuscript. PKA, NLA and RSM accessed and verified the underlying statistical calculations. PKA and RSM supervised the project. All authors read and approved the final version of the manuscript.

## Data sharing statement

All data are available in the main text or the Supplementary materials. Further clinical information supporting the findings of this study are available to those eligible upon request from the corresponding authors. Data will be stored for a minimum of 7 years.

## Declaration of interests

SOR is the chair of the Young Epilepsy Section, ILAE, and has received consulting fees from Biopas-UCB, support for attending meetings and/or travel from Mythotherapies, and speaker fees from Abbott, LivaNova, Sanofi, Biopas-UCB and Nutricia. MT has received consulting fees from Biomarin, support for attending meetings and/or travel from Biomarin and Jazz Pharmaceuticals, and participated in Data Safety Monitoring Boards or Advisory Boards for Biocodex. SA is the deputy editor of Epilepsia, and has received consulting fees from UCB, Xenon, Encoded Therapeutics, EISAI, Stoke, Proveca, speaker fees from Biocodex, EISAI, Jazz Pharmaceuticals, Neuraxpharm, Nutricia and UCB and participated in Data Safety Monitoring Boards or Advisory Boards for GRIN Therapeutics. JK has received consulting fees from Biomarin, support for attending meetings and/or travel from Biomarin and Jazz Pharmaceuticals, and participated in Data Safety Monitoring Boards or Advisory Boards for Biocodex. SW has received consulting fees from UCB, Knopp Biosciences, Encoded Therapeutics, Roche, support for attending meetings and/or travel from Angelini Pharma, and participated in Data Safety Monitoring Boards or Advisory Boards for Angelini Pharma and Xenon Pharmaceuticals. NS has received consulting fees from Biomarin, support for attending meetings and/or travel from Biomarin and Jazz Pharmaceuticals, and participated in Data Safety Monitoring Boards or Advisory Boards for Biocodex. PB has received consulting fees from LivaNova, EISAI, Jazz Pharmaceuticals, Angelini Pharma and support for attending meetings and/or travel from Angelini Pharma and EISAI. RSM has received consulting fees from UCB, Orion, Saniona, Immedica and Atalanta, and speaker fees from EISAI, Angelini Pharma, Jazz Pharmaceuticals, Orion and UCB. PC is Executive Vice President, Research at the company Saniona in Denmark. The remaining authors declare no competing interests.

## Appendix ASupplementary data

Supplementary Table S1 Supplementary Table S1. Detailed clinical information for all individuals and functional data.
